# multi‐dice: r package for comparative population genomic inference under hierarchical co‐demographic models of independent single‐population size changes

**DOI:** 10.1111/1755-0998.12686

**Published:** 2017-05-30

**Authors:** Alexander T. Xue, Michael J. Hickerson

**Affiliations:** ^1^ Department of Biology: Subprogram in Ecology, Evolutionary Biology, and Behavior City College and Graduate Center of City University of New York New York NY USA; ^2^ Division of Invertebrate Zoology American Museum of Natural History New York NY USA

**Keywords:** aggregate site frequency spectrum, approximate Bayesian computation, comparative phylogeography, population genetics software, random forest

## Abstract

Population genetic data from multiple taxa can address comparative phylogeographic questions about community‐scale response to environmental shifts, and a useful strategy to this end is to employ hierarchical co‐demographic models that directly test multi‐taxa hypotheses within a single, unified analysis. This approach has been applied to classical phylogeographic data sets such as mitochondrial barcodes as well as reduced‐genome polymorphism data sets that can yield 10,000s of SNPs, produced by emergent technologies such as RAD‐seq and GBS. A strategy for the latter had been accomplished by adapting the site frequency spectrum to a novel summarization of population genomic data across multiple taxa called the aggregate site frequency spectrum (aSFS), which potentially can be deployed under various inferential frameworks including approximate Bayesian computation, random forest and composite likelihood optimization. Here, we introduce the r package multi‐dice, a wrapper program that exploits existing simulation software for flexible execution of hierarchical model‐based inference using the aSFS, which is derived from reduced genome data, as well as mitochondrial data. We validate several novel software features such as applying alternative inferential frameworks, enforcing a minimal threshold of time surrounding co‐demographic pulses and specifying flexible hyperprior distributions. In sum, multi‐dice provides comparative analysis within the familiar R environment while allowing a high degree of user customization, and will thus serve as a tool for comparative phylogeography and population genomics.

## 1 INTRODUCTION

Population genetics has experienced an increasing interest in quantifying shared and idiosyncratic attributes across demographic histories from multiple independent taxa to address questions regarding wide‐scale biogeographic, ecological and evolutionary responses to climate and landscape changes, an endeavour commonly referred as comparative phylogeography (Arbogast & Kenagy, 2001; Avise, 2000; Hewitt, 1996, 2000; Hickerson et al., 2010; Papadopoulou & Knowles, 2016; Taberlet, Fumagalli, Wust‐Saucy, & Cosson, 1998). These comparative studies can be especially informative about how key environmental and organismal features (Carnaval, Hickerson, Haddad, Rodrigues, & Moritz, 2009; Carstens, Gruenstaeudl, & Reid, 2016; Fouquet et al., 2012; He et al., 2016; Kautt, Machado‐Schiaffino, & Meyer, 2016; Luo et al., 2015; Nadachowska‐Brzyska, Li, Smeds, Zhang, & Ellegren, 2015; Papadopoulou & Knowles, 2015; Qu et al., 2015; Rougemont et al., 2017; Smith et al., 2014; Stone et al., 2012; Wood et al., 2013) and selective forces (Boyko et al., 2010; Frantz et al., 2015; Gignoux, Henn, & Mountain, 2011; Hohenlohe et al., 2010; Poh, Domingues, Hoekstra, & Jensen, 2014; Rougeux, Bernatchez, & Gagnaire, 2016) affect patterns of shared and idiosyncratic histories. One approach in such investigations is to exploit multi‐taxa genetic data for comparative demographic inference under a hierarchical model, whereby hyperparameters govern the variability of a certain demographic parameter across taxa, while all other nuisance demographic parameters freely vary per each taxon (Beaumont, 2010; Hickerson, Dolman, & Moritz, 2006). In contrast to assembling results from independently performed inferential analyses to qualitatively compare demographic histories post hoc, this strategy permits explicit hypothesis testing and inference of multi‐taxa questions, as well as allows for gains in statistical power via the borrowing strength achieved from combining exchangeable data sets (Congdon, 2001; Gelman, Carlin, Stern, & Rubin, 2003; Qian et al., 2004), demonstrated previously via simulations (Xue & Hickerson, 2015).

Originally developed for single‐locus DNA data sets easily collected from multiple taxa (Burbrink et al., 2016; Hickerson, Stahl, & Takebayashi, 2007; Ornelas et al., 2013), this methodology has been extended to accommodate SNP data sets derived via recently emerging technologies such as RAD‐seq and GBS, thereby improving inferential resolution through vastly greater sampling of independent gene tree histories across genomes from multiple taxa (Xue & Hickerson, 2015). This has been accomplished by exploiting the aggregate site frequency spectrum (aSFS), which has been established to contain signal of variability in demographic histories across taxa. Producing an aSFS involves creating single‐population site frequency spectra (SFS) independently across taxa and combining these according to a standardized re‐ordering procedure based on relative proportions of total SNPs per allele frequency class. This protocol therefore does not require sites to be homologous between taxa and in turn allows data to be collected across distantly‐related taxa (more details about data preparation given in Implementation section). Construction of the aSFS can then be applied to coalescent simulations produced under a hierarchical co‐demographic model that treats taxa as independent, unidentified and exchangeable units, and coupled with a statistical framework such as approximate Bayesian computation (ABC) to make comparative multi‐taxa inference (Prates et al., 2016; Xue & Hickerson, 2015). This simulation approach could potentially be modified with other techniques, including machine learning algorithms such as random forest (RF) (Díaz‐Uriarte & Alvarez de Andrés, 2006; Pudlo et al., 2016; Strobl, Boulesteix, Zeileis, & Hothorn, 2007; Svetnik et al., 2003) and partial least squares regression (PLS) (Boulesteix & Strimmer, 2007; Wegmann, Leuenberger, & Excoffier, 2009). To elaborate, RF involves constructing decision trees based on “training” simulations to form a classification or regression scheme that subsequently can be applied to observed data, and PLS entails maximizing the variance explained in response variables in a manner similar to principal component analysis, which can be employed as a transformation procedure to potentially mediate high dimensionality of correlated summary statistics, such that inherently exists among aSFS bins. Alternatively, the aSFS could be deployed within a composite likelihood optimization (CL) framework, a statistical approach commonly used for demographic inference based on SFS data (Bustamante, Wakeley, Sawyer, & Hartl, 2001; Excoffier, Dupanloup, Huerta‐Sánchez, Sousa, & Foll, 2013; Gutenkunst, Hernandez, Williamson, & Bustamante, 2009; Lukic & Hey, 2012; Sawyer & Hartl, 1992).

The aSFS enables researchers to exploit data produced by next‐generation sequencing to explore a variety of hypotheses that relate climatic and landscape changes with the evolution and demographic histories of biotic assemblages through hierarchical co‐demographic modelling. Here we make this analytical pipeline available as the r package multi‐dice (*Multi*ple Taxa *D*emographic *I*nference of *C*ongruency in *E*vents). To demonstrate and explore implementation of multi‐dice, we conducted a series of simulation studies that summarize an expanded set of options within our aSFS approach, including: (i) employing RF as an additional inferential tool; (ii) enforcing a “buffer” on prior space such that co‐demographic events have an a priori minimal difference in time from each other; (iii) truncating the hyperprior range for improved hyperparameter estimation.

## 2 MATERIALS AND METHODS

### 2.1 Hierarchical co‐demographic model

**Figure 1:**
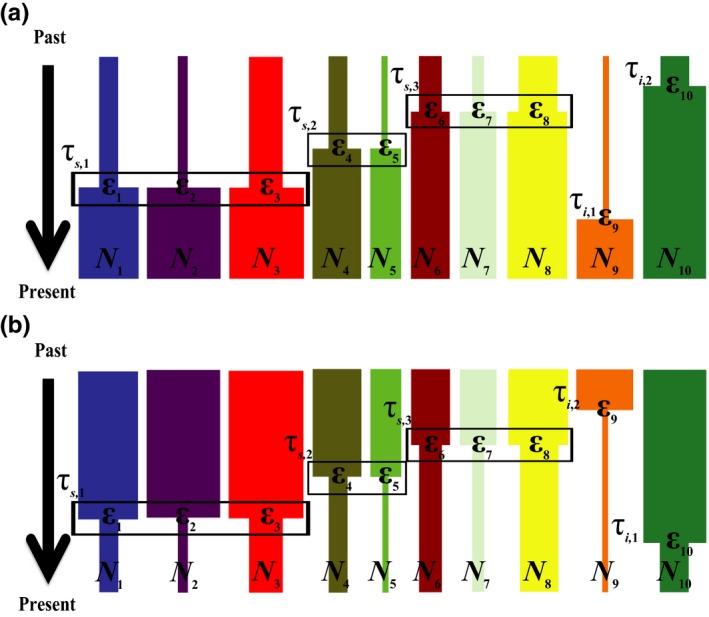
Hierarchical co‐demographic models. (a) Example instantaneous co‐expansion model. (b) Example instantaneous co‐contraction model. Both models are such that eight of the ten taxa are assigned to three synchronous co‐demographic pulses (ѱ = 3; ζ_*T*_ = 0.8), with the first pulse containing three taxa (ζ_1_ = 0.3), the second pulse containing another two taxa (ζ_2_ = 0.2) and the third pulse containing yet another three taxa (ζ_3_ = 0.3). Pulse 1 occurs at the most recent time (τ_*s*,1_), pulse 2 occurs at the intermediate time (τ_*s*,2_), and pulse 3 occurs at the most ancient time (τ_*s*,3_). The remaining two taxa are then behaving idiosyncratically in time from all other taxa (τ_*i*,1_ and τ_*i*,2_). Each taxon is allowed nuisance demographic parameter draws independent from each other ({ε_1_, …, ε_10_} and {*N*_1_, …, *N*_10_})

Our hierarchical co‐demographic model consists of *n* taxa, which refer to independent panmictic populations with no assumption of or requirement for recent shared ancestry (Mazet, Rodríguez, Grusea, Boitard, & Chikhi, 2016), randomly assigned to Ψ instantaneous expansion (Figure 1a) or contraction (Figure 1b) times. Of the Ψ times, there are ψ times corresponding to synchronous pulse events that involve at least two taxa, and σ times corresponding to idiosyncratic events ungrouped from any pulses with only a single taxon, such that Ψ = ψ + σ (Table 1). The proportion of *n* taxa assigned to any of the ψ pulses is represented by ζ_*T*_, the proportion of *n* taxa belonging to each of the ψ pulses is described by the associated hyperparameter vector ζ_*s*_ = {ζ_1_, …, ζ_ψ_}, and the proportions of *n* taxa across all Ψ events are indexed by the vector ζ = {ζ_*s*_, ζ_*i*,1_, …, ζ_*i*,σ_}. Here, ζ_*T*_ is a single proportion value that ranges from 0.0 to 1.0 and equals the total sum of ζ_*s*_ (i.e., ζ_*T*_ = $\sum_{j=1}^{\psi }\zeta_{j}$ when ψ > 0), and both ζ_*s*_ and ζ are hyperparameter vectors that index proportion values across events. Specifically, each of ψ elements within the vector ζ_*s*_ ranges from 2/*n* to 1.0, and ζ comprises of ζ_*s*_ as well as each ζ_*i*_ element = 1/*n*. The proportion ζ_*T*_ and proportions within the vector ζ_*s*_ may be converted to numbers of taxa *S*_*T*_ = ζ_*T*_ × *n* and *S *= ζ_*s*_ × *n*, respectively. Synchronous pulse times are indexed in the vector τ_*s*_ = {τ_*s*,1_, …, τ_*s*,ψ_}, whereas idiosyncratic times are indexed in the vector τ_*i*_ = {τ_*i*,1_, …, τ_*i*,σ_}, with both vectors arranged in ascending order from most recent to oldest. To clarify, synchronous pulses are indexed by the temporal order established by τ_*s*_ = {τ_*s*,1_, …, τ_*s*,ψ_}, which thus determines the order of ζ_*s*_ such that ζ_1_ pertains to the most recent pulse and ζ_ψ_ reflects the most ancient. In the case of Ψ = ψ and σ = 0, accordingly ζ_*T*_ = 1.0 such that all taxa are assigned to one of ψ synchronous pulses with no temporally idiosyncratic taxa. On the other extreme, when Ψ = σ = *n* and ψ = 0, accordingly ζ_*T*_ = 0.0 with zero elements in the associated ζ_*s*_ vector such that there are no synchronous pulses with all taxa idiosyncratically experiencing population size change across σ different times. Other taxon‐specific demographic parameters include each taxon's ratio of size change from the ancestral effective population size to current effective population size is indexed by the vector ε = {ε_1_, …, ε_*n*_} and each taxon's current effective population size indexed by the vector *N *= {*N*_1_, …, *N*_*n*_}. Additionally, population size change times may be indexed to coincide with the taxa arrangement of ε and *N* such that τ = {τ_1_, …, τ_*n*_} (Table 1).

**Table 1:** Glossary of hyperparameters, parameter summaries, and parameters

Hyper/parameter (*summary*) symbol	Details
Ψ	Number of total events; hyperparameter that directly governs ζ and in turn governs τ; Ψ = ψ + σ
ψ	Number of synchronous pulse events; hyperparameter that directly governs ζ_*s*_ and in turn governs τ_*s*_
ζ_*T*_	Total proportion of taxa belonging to any of ψ pulses; ranges from 0.0 to 1.0; ζ_*T*_ = $\sum_{j=1}^{\psi }\zeta_{j}$ when ψ* *≥ 1
ζ	Vector of proportions of taxa belonging to each event, thus including ζ_*s*_, ordered such: {ζ_*s*_, ζ_*i*,1_, …, ζ_*i*,σ_}, with each ζ_*i*_ element = 1/*n*; hyperparameter that directly governs τ
ζ_*s*_	Vector of proportions of taxa belonging to each pulse {ζ_1_, …, ζ_ψ_}, ordered from most recent to most ancient; hyperparameter that directly governs τ_*s*_; each element ranges from 2/*n* to 1.0
ζ_*i*_	An element of ζ or ζ_*s*_ as the index *j* iterates from 1 to Ψ or ψ, respectively
*S*_*T*_	Conversion of ζ_*T*_ to numbers of taxa by ζ_*T*_ * *n*;* n *=* S*_*T*_ + σ
*S*	Conversion of ζ_*s*_ to numbers of taxa by ζ_*s*_ * *n*
σ	Number of idiosyncratic events, and thus idiosyncratic taxa as well; determines length of τ_*i*_
τ	Vector of times across *n* taxa in units of number of generations that corresponds to ε and *N*
τ_*s*_	Vector of synchronous pulse times corresponding to ζ_*s*_ and thus in coinciding order from most recent to most ancient
τ_*i*_	Vector of idiosyncratic pulse times and similarly ordered from most recent to most ancient
ε	Vector of nuisance size change magnitudes in units of ratio from ancestral *N*_*E*_ to current *N*_*E*_; corresponds to τ and *N*; though not explored here, within multi‐dice, this parameter could be hyperparameterized by Ψ/ψ and ζ/ζ_*s*_ instead of or in complement to τ
*N*	Vector of nuisance *N*_*E*_; corresponds to τ and ε; though not explored here, within multi‐dice, this parameter could be hyperparameterized by Ψ/ψ and ζ/ζ_*s*_ instead of or in complement to τ
*n*	Total number of taxa in data set
β	Pulse buffer value, in units of number of generations, between pulses and thereby modifying the τ prior; though not explored here, if ε or *N* were hyperparameterized, those pulses could be accordingly buffered, and β could be delineated by β_ε_ and β_*N*_, respectively
Ω_τ_	Dispersion index of τ, or Var(τ)/*E*(τ), a parameter summary describing temporal variation among taxa for which there is strong inferential power; though not done here, could be calculated for ε and *N* as well

When implementing this co‐demographic model for comparative demographic inference, there exists flexibility in the hierarchical parameterization, with several options available in multi‐dice. One such option, similar to the approach described in Chan, Schanzenbach, and Hickerson (2014) and Xue and Hickerson (2015), is to constrain the hyperparameter ψ to the values within the set {0, 1} and condition Ψ and σ on the hyperparameter ζ_*T*_, which freely varies according to the hyperprior distribution *P*(ζ_*T*_). This allows scenarios of complete idiosyncrasy, absolute synchrony within a single pulse, and intermediate degrees of synchronicity belonging to one pulse with remaining taxa temporally idiosyncratic. Here, ζ_1_ is the only element possible in ζ_*s*_ whereby ζ_*T*_ = ζ_1_ when ψ = 1 and ζ_*T*_ = 0.0 when ψ = 0, resulting in the joint posterior distribution *P*(ζ_*T*_, τ, ε, *N* | Data) ∝ *P*(Data | ζ_*T*_, τ, ε, *N*) *P*(ε, *N*) *P*(τ | Ψ, σ, ζ_*T*_) *P*(Ψ, σ | ζ_*T*_) *P*(ζ_*T*_ | ψ < 1). The values for Ψ and σ are then determined by Ψ = 1 + *n *− *S*_*T*_ (when ψ* *=* *1) and σ* *=* n *− *S*_*T*_, respectively.

An alternative scheme is to randomly assign the proportions of *n* taxa to Ψ times according to the hyperprior distribution for the vector ζ, which is conditional on the hyperprior distribution of Ψ, with ψ and σ accordingly conditional on *P*(ζ | Ψ) and *P*(Ψ). This leads to the joint posterior distribution *P*(Ψ, ζ, τ, ε, *N* | Data) ∝ *P*(Data | Ψ, ζ, τ, ε, *N*) *P*(ε, *N*) *P*(τ | Ψ, ζ, ψ, σ) *P*(ψ, σ | Ψ, ζ) *P*(ζ | Ψ) *P*(Ψ). The values for ψ and σ are then determined by the number of Ψ draws for ζ that are above and equal to 1/*n*, respectively, yielding the so‐called Chinese restaurant process (Aldous, 1985; Blei, Griffiths, Jordan, & Tenenbaum, 2003) that is similarly applied in *msBayes* (Hickerson et al., 2007; Huang, Takebayashi, Qi, & Hickerson, 2011). Similarly, a third scheme is to condition the hyperprior distribution for the vector ζ_*s*_, which must have a lower bound greater than 1/*n*, on the hyperprior distribution of ψ, with Ψ and σ accordingly conditional on *P*(ζ_*s*_ | ψ) and *P*(ψ), such that the joint posterior distribution is *P*(ψ, ζ_*s*_, τ, ε, *N* | Data) ∝ *P*(Data | ψ, ζ_*s*_, τ, ε, *N*) *P*(ε, *N*) *P*(τ | ψ, ζ_*s*_, Ψ, σ) *P*(Ψ, σ | ψ, ζ_*s*_) *P*(ζ_*s*_ | ψ) *P*(ψ). The values for Ψ and σ are then determined by Ψ = ψ + *n* − *S*_*T*_ and σ = *n* − *S*_*T*_, respectively. Optionally, for each possible value in the ψ hyperprior, the associated ζ_*s*_, Ψ and σ values may be fixed to specified values rather than allowed to vary.

### 2.2 Simulation experiments

We conducted a series of in silico experiments to quantify accuracy and bias for various inferential frameworks and hierarchical co‐demographic modelling variants. Data were simulated under known hyperparameter and parameter values with the coalescent simulator fastsimcoal version 2.5 (Excoffier et al., 2013). To directly generate single‐population folded SFS, the FREQ setting was enabled assuming a set number of independent genealogies per SFS, which was treated as an approximation for the number of SNPs sampled and differed between experiments. Each SFS contained 20 haploid samples, only polymorphic bins and proportional SNP frequencies rather than total SNP counts. Per individual simulation, a set of 10 SFS corresponding to *n *= 10 populations was converted to a single aSFS summary vector following Xue and Hickerson (2015). Simulation reference tables composed of hyperparameter and parameter values randomly drawn from their respective hyperprior and prior distributions and their corresponding aSFS summaries were separately produced for each hierarchical co‐demographic model variant and read into the R environment with the r package bigmemory to perform hierarchical RF regression (hRF) and hierarchical ABC (hABC) under the simple rejection algorithm against pseudo‐observed data sets (PODs). PODs were produced under one of two methods, either independently from the reference table or using the “leave‐one‐out” cross‐validation procedure. In brief, the “leave‐one‐out” procedure involves iteratively treating a single randomly selected simulation from a reference table as a POD and conducting inference using the remaining simulations (Csilléry, François, & Blum, 2012). For each inferential application, Pearson's *r* correlation and root mean squared error (*RMSE*) were calculated from estimated values against true POD values.

### 2.3 Testing inferential frameworks

In addition to hRF and hABC, we coupled these frameworks with transformation of the aSFS by PLS as well as evaluated the performance of hierarchical CL (hCL). To compare these inferential strategies, per each of the two hierarchical co‐demographic models of co‐expansion and co‐contraction (Figure 1), 100 aSFS PODs were simulated under the hyperprior distribution of ψ ~ *U*{0, 5} while permitting idiosyncratic taxa such that ζ_*T*_ was allowed to vary from 0.0 to 1.0. These PODs were consistently utilized to independently estimate ψ across each inferential approach. A reference table of 1,000,000 simulated aSFS was likewise produced per model under the same specification as the PODs (Supporting Information). For hRF, using the r package randomforest (Liaw & Wiener, 2002), a total of 1,000 decision trees, with the default maximum of 33 variables randomly sampled as candidates at each tree split and from 10 trees per each of 100 cycles of randomly subsampling 1,000 simulations per ψ (for a total of 6,000 simulations) with replacement after each cycle, were built per reference table to capture variation in ψ and leveraged to predict ψ for each corresponding POD using the *predict()* function. For hABC, using the function *abc()* from the r package abc (Csilléry et al., 2012), accepted tolerance levels of 0.0050, 0.0010 and 0.0005 were executed per POD against the corresponding reference table, and the mean, median and mode of the according posterior distributions were calculated for point estimates of ψ.

For PLS, the *plsr()* function in the r package pls (Mevik & Wehrens, 2007) was applied to a random subset of 10,000 simulations against variation in ψ per reference table. The PLS for each reference table was subsequently utilized to transform the remaining 990,000 simulations and corresponding PODs into as many component values as needed to explain ≥95% of the total variance in the original summary statistics. The same hRF and hABC protocols were then executed on the remaining transformed reference tables. For hCL, a custom pipeline that calls *dadi* to calculate the expected SFS (Gutenkunst et al., 2009) and incorporates the multinomially distributed CL equation utilized in fastsimcoal2 (Excoffier et al., 2013) and the BFGS optimization algorithm (Liu & Nocedal, 1989) was implemented (Supporting Information).

### 2.4 Pulse buffer on prior space

Estimation of ψ or Ψ can be problematic as it does not necessarily correlate well with true temporal variability in co‐demographic events. For example, a large number of synchronous events closely clustered in time would signify a high ψ value yet have low temporal variability, whereas a history with two synchronous co‐demographic events that are far apart in time would yield a lower ψ value (ψ = 2) but with higher variance in time. As is the case with previous implementations of hierarchical co‐demographic models (Hickerson et al., 2014), this inconsistency can hinder the ability to capture meaningful signal of ψ contained within the aSFS. To improve ψ estimation, we deployed a user‐defined temporal pulse buffer that defines a minimal threshold of time β surrounding each co‐demographic event such that for each *j*th event, all other co‐demographic events occur outside a τ_*j*_ ± β window. Mechanistically, this involves sequentially modifying the prior distribution with every subsequent τ draw, with final assignment of {τ_*s*,1_, …, τ_*s*,ψ_} in ascending order such that τ_*s*,1_ is the most recent and τ_*s*,ψ_ is the most ancient. For example, given a simulation with values ψ = 2, τ ~ *U*{10,000, 1,000,000} and β* *=* *20,000, if the first τ_*s*_ draw is 100,000 generations, then the second τ_*s*_ draw would be from the set *U*{10,000, 79,999} ∪ *U*{120,001, 1,000,000}; and if the second τ_*s*_ draw is 15,000 generations, then {τ_*s*,1_, τ_*s*,2_} is assigned such that τ_*s*,1_ = 15,000 and τ_*s*,2_ = 100,000. Importantly, a limit on the allowable number of buffered co‐demographic events is imposed by the total τ prior distribution across these events and the magnitude of β.

### 2.5 Testing pulse buffer on prior space

To gauge how β impacts hyperparameter estimation, two reference tables with β = 0 generations and β = 30,000 generations were generated. In the special case of ψ = 0 for the β = 30,000 reference table, β was reduced to 10,000 due to the constraint from the τ prior range and to allow more flexibility in the temporal dispersion for the total idiosyncrasy scenario. Both reference tables contained 100,000 aSFS simulations of instantaneous co‐expansion (Figure 1a) per value of ψ ~ *U*{0, 5} for a total of 600,000 simulations each. For simplicity, idiosyncratic taxa were not permitted and ζ_*T*_ = 1.0 was evenly distributed across the vector ζ_*s*_ for each value of ψ > 0 (Table 2). Importantly, to accommodate the special case of ψ = 0, which is equivalent to Ψ = 10, whereas all other values of ψ result in Ψ = ψ, ψ values were converted to Ψ for estimation purposes. Single‐population SFS were generated from 5,000 independent genealogies and according to the prior distributions τ ~ *U*{5,000, 250,000} (in units of number of generations), ε ~ *U*(0.01, 0.10) and *N* ~ *U*{50,000, 250,000}.

**Table 2:** ζ_*s*_ values given even distribution of ζ_*T*_ = 1.0 for each value of ψ > 0

ψ value	ζ_*s*_ values
ψ = 1	ζ_1_ = 1.0
ψ = 2	{ζ_1_, ζ_2_} = 0.5
ψ = 3	{ζ_1_, ζ_2_, ζ_3_} = {0.4, 0.3, 0.3} (in random order per simulation)
ψ = 4	{ζ_1_, ζ_2_, ζ_3_, ζ_4_} = {0.3, 0.3, 0.2, 0.2} (in random order per simulation)
ψ = 5	{ζ_1_, ζ_2_, ζ_3_, ζ_4_, ζ_5_} = 0.2

The “leave‐one‐out” cross‐validation procedure was performed on each reference table for hRF and hABC hyperparameter estimation of Ψ. This followed the same specifications as for testing inferential frameworks, except the function *cv4postpr()* from the r package abc (Csilléry et al., 2012) was deployed for hABC model selection and the selected PODs were collectively removed from the reference table for hRF cross‐validation. For every reference table, 20 “leave‐one‐out” POD iterations per Ψ value yielded a total of 120 PODs, and an accepted tolerance level of 0.0025 resulting in 1,500 total retained simulations. Each discrete value of Ψ was treated as a separate model, although the numeric values of Ψ were exploited to determine the mean and median of the model posterior distribution. Furthermore, the function *cv4abc()* from the r package abc was utilized for hABC parameter summary estimation cross‐validation of Ω_τ_ (Var(τ)/E(τ), or dispersion index of τ) and E(τ), following the same specifications as hABC model selection cross‐validation, across 50 total “leave‐one‐out” POD iterations per reference table. In addition, another cross‐validation experiment was conducted on the β = 30,000 reference table with PODs from the β = 0 reference table. The same protocols for Ψ hyperparameter estimation with hRF and hABC model selection and Ω_τ_ and E(τ) parameter summary estimation with hABC were performed here, except the functions *postpr()* and *abc()* from the r package abc were employed for hABC hyperparameter and parameter summary estimation, respectively. This particular experiment can demonstrate the power of parameterizing clustered events together using a buffer even though real data are not under such constrictions.

### 2.6 Testing truncated hyperprior range

To explore the effect of decreasing hyperprior upper bounds on ψ, we took subsets of the aforementioned β = 30,000 reference table in order to construct new reference tables that corresponded to ψ ~ *U*{0, 5}, ψ ~ *U*{0, 4}, ψ ~ *U*{0, 3}, ψ ~ *U*{0, 2} and ψ ~ *U*{0, 1}, respectively (Table 3). By cross‐validating these subset reference tables given reduced hyperprior ranges, we can assess the discriminatory power of ψ values under differing hyperparameterizations. In this exploration, cross‐validation was restricted to only “leave‐one‐out” Ψ estimation via hRF and hABC model selection per reference table, following the previously outlined specifications for testing the pulse buffer.

**Table 3:** Specifications of subset reference tables for truncating hyperprior range simulation experiment

Subset reference table hyperprior	Total simulations (based on 100,000 per ψ value)	Total PODs (based on 20 per ψ value)	Total sub‐sampled simulations for each cycle of 10 hRF decision trees (based on 1,000 per ψ value)	Remaining simulations for hRF sub‐sampling once PODs removed	hABC accepted tolerance level (leading to 1,500 retained simulations)
ψ ~ *U*{0, 5}	600,000	120	6,000	599,880	0.00250
ψ ~ *U*{0, 4}	500,000	100	5,000	499,900	0.00300
ψ ~ *U*{0, 3}	400,000	80	4,000	399,920	0.00375
ψ ~ *U*{0, 2}	300,000	60	3,000	299,940	0.00500
ψ ~ *U*{0, 1}	200,000	40	2,000	199,960	0.00750

## 3 RESULTS/DISCUSSION

### 3.1 Testing inferential frameworks

The inferential frameworks that demonstrated the highest accuracy and precision in estimating ψ were hRF (*r *= .600–.807, *RMSE* = 1.77–2.22) and hABC mean estimates (*r *= .500–.802, *RMSE* = 1.76–2.41; Table 4). Interestingly, there was improvement in estimating ψ with hRF compared to hABC, as well as estimating ψ under the co‐contraction model in contrast to the co‐expansion model. Importantly, PLS transformation worsened performance considerably in nearly all cases, suggesting that it is not a viable option within this context, especially considering its large memory requirements. Furthermore, hCL performed very poorly, which likely can be attributed to insufficient sampling of the vast multi‐taxa and multi‐level parameter space by hCL's intensive optimization approach. The hCL implementation that we used could potentially be improved, for example, using a different exploration tactic for nuisance parameters and more independent optimization replicates. Indeed, accurate estimates should be achievable provided an intensive sampling of the parameter space. Nonetheless, given finite computational resources, the quite poor performance here heavily suggests that likelihood approaches generally are not advised for our set of hierarchical co‐demographic models, unlike other inferential applications on single‐taxon SFS data sets (Excoffier et al., 2013; Gutenkunst et al., 2009; Lukic & Hey, 2012). This is especially relevant for large data sets considering that computational requirements scale unfavourably with increasing taxa membership due to the growth of hyperparameter space. On the other hand, the stronger performances of hRF and hABC suggest that these are sensible inferential choices to pair with the aSFS. Moreover, they offer computational and statistical advantages such as ease in parallelizing simulation efforts, minimal effort needed to exploit a single reference table for conducting multiple empirical estimates as is done in a cross‐validation analysis with PODs, and flexibility in specifying nuisance parameters.

**Table 4:** Results for testing inferential frameworks simulation experiment

	Instantaneous co‐expansion		Instantaneous co‐contraction	
	*r*	*RMSE*	*r*	*RMSE*
hRF prediction of Ψ	.600	2.22	.807	1.77
hRF coupled with PLS prediction of Ψ	.469	2.44	.831	1.73
hABC hyperparameter estimation of Ψ				
tol. = 0.0050				
Mean	.500	2.41	.800	1.77
Median	.426	2.85	.733	2.03
Mode	.413	3.19	.602	2.67
tol. = 0.0010				
Mean	.534	2.36	.800	1.77
Median	.428	2.85	.735	2.03
Mode	.427	3.05	.631	2.53
tol. = 0.0005				
Mean	.547	2.34	.802	1.76
Median	.495	2.71	.758	1.95
Mode	.481	2.94	.666	2.40
hABC coupled with PLS hyperparameter estimation of Ψ				
tol. = 0.0050				
Mean	.323	2.75	.612	2.83
Median	.251	2.75	.392	2.99
Mode	.234	2.75	.301	2.99
tol. = 0.0010				
Mean	.384	2.67	.641	2.61
Median	.267	2.74	.466	2.82
Mode	.277	2.76	.385	2.88
tol. = 0.0005				
Mean	.402	2.64	.665	2.52
Median	.221	2.77	.457	2.84
Mode	.202	2.85	.397	2.90
hCL optimization of Ψ	.027	4.10	.259	3.49

### 3.2 Improved Ψ estimation with pulse buffer β and truncated hyperprior

According to our cross‐validation experiments, there is greater reliability in estimating Ψ with both hRF and hABC by incorporating a pulse buffer on τ prior space (Table 5). Moreover, when incorporating the β = 30,000 reference table against PODs simulated under β = 0, there was improved Ψ estimation for both hRF and hABC in comparison with the “leave‐one‐out” cross‐validation on the β = 0 reference table. Additionally, buffering appears to benefit hyperparameter estimation without substantially affecting hABC estimation of parameter summaries Ω_τ_ and E(τ). Notably, hRF again outperformed hABC in Ψ estimation, although this was minimal.

**Table 5:** Results for pulse buffer on prior space simulation experiment

	β = 0 generations		β = 30,000 generations		PODs: β = 0 reference table: β = 30,000	
	*r*	*RMSE*	*r*	*RMSE*	*r*	*RMSE*
hRF prediction of Ψ	.609	2.32	.758	1.91	.666	2.26
hABC model selection of Ψ						
Mean	.600	2.37	.750	1.96	.617	2.43
Median	.557	2.65	.686	2.20	.596	2.71
Mode	.507	3.07	.722	2.18	.527	2.91
hABC parameter summary estimation of Ω_τ_						
Mean	.932	7555	.874	9750	.904	11009
Median	.886	12616	.860	11120	.905	11042
Mode	.846	13727	.889	12775	.826	15227
hABC parameter summary estimation of *E*(τ)						
Mean	.945	14550	.927	12539	.962	13072
Median	.920	14199	.946	11738	.962	12923
Mode	.915	15983	.949	12222	.957	13644

Better performance in Ψ estimation is apparent when truncated hyperprior ranges were employed, with ψ ~ *U*{0, 3} possibly the best compromise here between a more flexible hyperprior and greater accuracy (Table 6). This is perhaps unsurprising considering that there likely is decreasing identifiability between higher Ψ values, such that higher Ψ values are both quantitatively and qualitatively less distinguishable. For example, higher Ψ values may be expected to have more broadly overlapping Ω_τ_ values, and the difference between four and five pulses may be biologically less important than between one and two. The decreased accuracy in Ψ estimation at wider hyperprior ranges highlights that it is impractical to construct a model that distributes significant prior space across values that are statistically indistinguishable and not qualitatively or biologically meaningful (Massatti & Knowles, 2016; Rannala, 2015). Indeed, as with any statistical model, sensible prior distributions given data and model constraints ought to be established (Bertorelle, Benazzo, & Mona, 2010; Lopes & Beaumont, 2010), especially when considering efficiency with respect to a finite sampling of parameter space (Hickerson et al., 2014). In the case here of hierarchical co‐demographic models, rather than using Ψ or ψ, as well as other parameters such as τ, in an arbitrary manner to merely construct the model, it can instead be specified meaningfully to gain insight about the variability in demographic changes across taxa given the temporal scale of interest.

**Table 6:** Results for truncating hyperprior range simulation experiment

	ѱ ~ *U*{0, 1}		ѱ ~ *U*{0, 2}		ѱ ~ *U*{0, 3}		ѱ ~ *U*{0, 4}		ѱ ~ *U*{0, 5}	
	*r*	*RMSE*	*r*	*RMSE*	*r*	*RMSE*	*r*	*RMSE*	*r*	*RMSE*
hRF prediction of Ψ	.987	0.73	.897	1.79	.809	2.08	.756	2.07	.758	1.91
hABC model selection of Ψ										
Mean	.963	1.22	.901	1.79	.808	2.13	.754	2.10	.750	1.96
Median	.900	2.01	.830	2.35	.705	2.65	.711	2.37	.686	2.20
Mode	.900	2.01	.864	2.11	.811	2.16	.744	2.35	.722	2.18

## 4 IMPLEMENTATION

**Figure 2:**
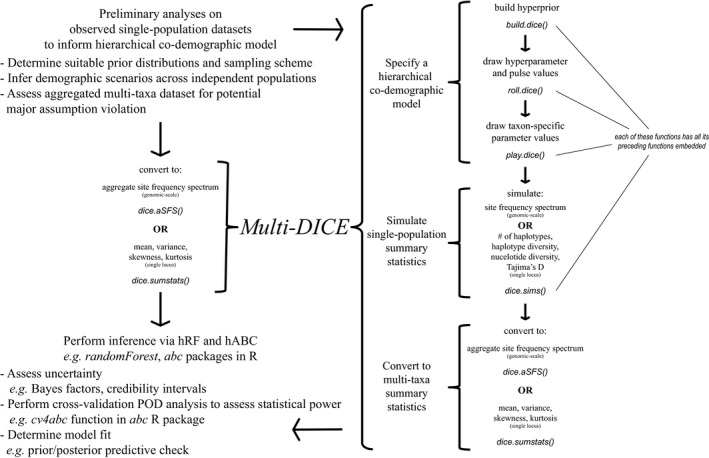
Flowchart of multi‐dice usage. multi‐dice accomplishes multi‐taxa co‐demographic inference under a hierarchical model through three major steps: model specification, single‐population simulation across multiple taxa and conversion of simulated data to multi‐taxa summary statistics. Hierarchical co‐demographic model specification is conducted across multiple functions in sequence, with preceding functions contained within successive functions. This sequential embedding of functions extends to *dice.sims()*, allowing the entire model specification process to be performed concurrently with data simulation. Simulated data can then be converted to multi‐taxa summary statistics by either *dice.aSFS()* or *dice.sumstats()*, depending on the data type. Additionally, these functions can be applied to empirical data as well. To clarify, only two multi‐dice functions/command lines, *dice.sims()* and *dice.aSFS()*/*dice.sumstats()*, are needed for simplest usage to construct a reference table of multi‐taxa summary statistics under a hierarchical co‐demographic model. This reference table can then be exploited in a downstream software program for hRF or hABC purposes, where appropriate statistical practices should be used to examine robustness and fit. Importantly, exploratory analyses should be performed on the empirical data prior to deploying multi‐dice to better guide its usage, for example, to determine sensible prior distributions and evaluate differences among taxa

Informed by our test of statistical frameworks, we offer multi‐dice as an r package, available on *github* with minimal dependencies (schoolmath, bigmemory and fbasics), to facilitate simulations under a hierarchical co‐demographic model with subsequent conversion to the aSFS or multi‐taxa mitochondrial summary statistics for inference within an hRF and/or hABC framework (Figure 2). Importantly, although inferential procedures are not conducted with multi‐dice itself, users are recommended to exercise sound and sensible statistical practices when analysing empirical data, such as evaluating uncertainty, implementing simulation‐based tests of robustness (Bertorelle et al., 2010) and assessing goodness of fit with techniques such as prior and posterior predictive checks (Gelman et al., 2003; Lemaire, Jay, Lee, Csilléry, & Blum, 2016). For multi‐dice, we employed bigmemory for efficient memory usage, necessary for the large simulation data requirements of hRF and hABC. Moreover, multi‐dice requires minimal effort to parallelize for greater computational efficiency. It is currently coded to call upon fastsimcoal2, which must be installed separately with its path specified in multi‐dice, for simulation purposes. We expressly chose fastsimcoal2 for its efficient coalescent‐based simulation of the SFS directly, growing user base, and approachable yet powerful modelling interface. However, given the architecture of the open source code, it is fairly straightforward to extend multi‐dice to usage with other simulators, including those that accommodate different forms of natural selection (Ewing & Hermisson, 2010; Kern & Schrider, 2016), or analytical calculations of the SFS (Kamm, Terhorst, & Song, 2017; Wakeley & Hey, 1997). Notably, although our focus here is on the aSFS and accordingly reduced representation data sets (e.g., SNPs, RAD‐seq, GBS), we acknowledge the great value in utilizing widely available mitochondrial/barcode‐type data (Burbrink et al., 2016) and therefore implement this functionality following the procedure in Chan et al. (2014).

### 4.1 R functions

multi‐dice is composed of the functions *build.dice()*,* roll.dice()*,* play.dice()*,* dice.sims()*,* dice.aSFS()* and *dice.sumstats()*. These functions are called to: (i) specify a hierarchical co‐demographic model; (ii) simulate under this model independent single‐population summary statistics (e.g., SFS) to accommodate each population with known parameter values drawn from user‐defined prior distributions and identical sampling specifications as the data; (iii) convert these independent single‐population summary statistics within both the simulations and empirical multi‐taxa data set into the aSFS or multi‐taxa single‐sequence summary statistics (Figure 2). This pipeline is carried over multiple functions to increase user customization and control, although the functions *build.dice()*,* roll.dice()* and *play.dice()* can together be called upon by *dice.sims()*, enhancing convenience by enabling consecutive function execution through a single command line. Additionally, a user may manually run any subset of these functions as antecedent functions are embedded and output may be piped into successive functions. For example, a user can construct hyperprior distributions using *build.dice()* and then immediately begin performing simulations through *dice.sims()*. After simulations are complete, either *dice.aSFS()* or *dice.sumstats()* is called to process the simulated and empirical data, which are then funnelled with the associated simulated parameter values into other software for inferential purposes, such as randomforest or abc in R. In its simplest operation then, multi‐dice can construct a reference table of simulated multi‐taxa summary statistic vectors produced under a hierarchical co‐demographic model for hRF and/or hABC in just two command lines, that is, *dice.sims()* and *dice.aSFS()*/*dice.sumstats()*.

### 4.2 Workflow

The function *build.dice()* is deployed first to construct hyperpriors across discrete hyperparameter values (i.e., Ψ, ψ, ζ_*T*_, ζ and ζ_*s*_), allowing the following distributions: (i) a discrete uniform hyperprior on Ψ or ψ, depending on how the associated ζ vector is specified, then for ζ_*T*_ within each discrete Ψ or ψ value, and finally across all combinations of the vector ζ or ζ_*s*_, respectively, within each discrete ζ_*T*_ value; (ii) a Dirichlet‐process hyperprior (Oaks, 2014) that weighs equally all allowable combinations of Ψ/ψ and ζ/ζ_*s*_; (iii) customized hyperprior distributions that may employ maximum and/or minimum value rules on ζ_*T*_, ζ and/or ζ_*s*_. To clarify for the uniform hyperpriors, each discrete Ψ or ψ value is first weighted with equal hyperprior probability, then all discrete ζ_*T*_ values are weighted equally per Ψ/ψ value, and finally every possible associated vector ζ/ζ_*s*_ is weighted equally per ζ_*T*_ value, thus underscoring that Ψ/ψ operates on another hierarchical level above ζ_*T*_, ζ and ζ_*s*_. Next, *roll.dice()* generates random draws from the hyperprior distributions as well as shared pulse values (e.g., {τ_*s*,1_, …, τ_*s*,ψ_}). Downstream to these steps is *play.dice()*, where taxon‐specific parameter values are generated and parameter summaries are calculated (e.g., Ω). Importantly, as both *roll.dice()* and *play.dice()* use the *sample()* function for random draws, each value in a user‐specified distribution is treated as unique even when values are repeated (e.g., ψ ∈ {0, 0, 0, 1, 2}), thus any discrete distribution (e.g., ln, gamma, beta) may be deployed for hyperpriors and priors. Together, *build.dice()*,* roll.dice()* and *play.dice()* specify the hierarchical co‐demographic model, as well as administer hyperparameter, parameter summary and taxon‐specific parameter draws given this model. Notably, data partitioning may be performed here (Prates et al., 2016), which allows heterogeneous specification of demographic scenarios (e.g., expansion, contraction), prior distributions, and data content and format (e.g., sampling of individuals, sampling time, polarization) across taxa within a data set; for example, data partitioning can accommodate a co‐demographic model of expanders mixed with contractors at a pre‐determined ratio.

In succession is *dice.sims()*, where fastsimcoal2 is called to simulate data independently per taxon. Here, heterogeneous generation times across taxa may be specified (Xue & Hickerson, 2015). Importantly, for genomic‐scale data, either the FREQ setting may be activated to directly generate SFS, or the SNP setting may be employed, which allows the option of using a mutation rate prior and thus monomorphic sites; for single‐locus data, the SNP setting is deployed. Simulated summary statistic vectors and associated hyperparameter draws, taxon‐specific parameter values and optional parameter summaries are outputted to a user‐specified directory as simple text files. The total number of outputted files equals the number of simulated taxa plus one file per hyperparameter, taxon‐specific parameter vector and parameter summary chosen for output. As aforementioned, all the functions described thus far can be implemented together automatically within *dice.sims()*, although independently calling functions may afford enhanced customization. Following *dice.sims()* is either *dice.aSFS()* or *dice.sumstats()*, depending on the data scale (i.e., genomic or single locus, respectively). For *dice.aSFS()*, the independent taxon‐specific SFS are rearranged into a single aSFS according to the procedure outlined in Xue and Hickerson (2015), and for *dice.sumstats()*, the first four moments (i.e., mean, variance, skewness and kurtosis) are calculated for each of the four summary statistics (i.e., number of haplotypes, haplotype diversity, nucleotide diversity and Tajima's *D*) of the single‐locus sequence block across the multiple taxa, for a total of 16 multi‐taxa summary statistics, following Chan et al. (2014). For both of these functions, the user specifies the directory containing the simulation files, with simple specification for multiple directories resulting from parallelized runs, and the subsequent conversion is outputted within R, enabling easy piping into an inferential package such as abc and/or writing to a simple text file. Importantly, these two functions can be applied to convert empirical data as well. Additionally, neither function calls upon any other multi‐dice functions and thus must be used in conjunction with at least *dice.sims()*.

Advantageously, data type is irrelevant in all functions until *dice.sims()*, for which the data type is easily specified in a single argument and there is no disparity in output format. Hence, hierarchical co‐demographic models can be specified with the same level of complexity and flexibility for single‐locus data as genomic‐scale data in multi‐dice. Furthermore, *dice.aSFS()* and *dice.sumstats()* operate analogously and have near identical arguments, resulting in equivalent procedures for both data types with negligible difference. This feature lends itself nicely to conveniently analysing both data types for the same system either consecutively or simultaneously.

### 4.3 Data sampling and processing

Although not directly handled by the multi‐dice package, we discuss here our recommendations for the practice of obtaining and preparing data. We emphasize that our methodology assumes population‐level sampling of multiple independent taxa, thus necessitating a sufficient number of samples per panmictic population (Robinson, Coffman, Hickerson, & Gutenkunst, 2014), which would depend on the temporal scale under investigation (Keinan & Clark, 2012). Importantly though, there is greater statistical resolution gained with increasing numbers of taxa (Chan et al., 2014; Xue & Hickerson, 2015), such that more emphasis should be placed on producing data sets with greater taxa representation rather than population‐level sampling. To achieve this, investigators can benefit from splitting species/complexes into multiple independent structured populations that are determined from a preliminary exploratory analysis (Frichot, Mathieu, Trouillon, Bouchard, & François, 2014; Patterson, Price, & Reich, 2006). This is especially important as lumping samples from multiple subdivided populations can result in strong bias when estimating population size changes (Mazet et al., 2016). While splitting indeed neglects shared ancestry, this problem may be negligible if isolation times are older than the co‐demographic events of interest. Relatedly, conducting a cross‐validation analysis across various sampling schemes, including both number of samples per taxon and number of taxa, prior to data collection and sequencing can be particularly informative of the proper sampling required for a given study (Bertorelle et al., 2010).

Greater statistical strength is gained with increasing taxa membership, but a strategy of indiscriminately adding taxa without consideration of specific characteristics can restrict researchers to testing generic hypotheses about assemblage‐level demographic responses to shared conditions (Papadopoulou & Knowles, 2016). In consideration of this, we encourage researchers to, whenever possible, delineate data sets based on guilds that share a trait of interest. This may include habitat preference (Papadopoulou & Knowles, 2015), biotic interaction such as parasitoid–host relationship (Stone et al., 2012) and other co‐evolutionary dynamics, or phylogenetic relatedness and taxonomic assignment (Burbrink et al., 2016).

We highlight here that the aSFS is capturing information within multiple independent structured populations, particularly size change history, through an aggregation of independent single‐population SFS vectors. This operates somewhat differently than a joint‐SFS or multi‐SFS across multiple related populations, which also contains information about divergence and migration from shared and fixed polymorphisms (Wakeley & Hey, 1997). By focusing on solely within‐population polymorphisms and being exploited to test hypotheses about size change history across taxa that may have experienced shared responses to climatic and habitat change while ignoring inter‐population relationships, the aSFS‐based approach simplifies the modelling, eliminates certain assumptions (e.g., topology, nature and duration of migration) and allows the option to directly test hypotheses across co‐distributed taxa. On a related note, if SNPs are pruned to one per locus to avoid linkage disequilibrium violations prior to constructing the observed SFS, and if SNP calls were conducted across populations, then fixed polymorphisms should be removed before pruning to maximize the total number of SNPs per population.

Although the focus here on the aSFS has been exclusively regarding SNPs, multi‐dice is capable of incorporating monomorphic sites and accordingly mutation rates. Importantly, considering how τ scales with *N*_*E*_ in a coalescent model, if prior distributions exceed one order of magnitude for both parameters, then nonidentifiable SFS at different parameter combinations may be produced by ignoring monomorphic sites, thus potentially inflating bias and inaccuracy. Hence, models that cannot have priors informed at least to this level may need to incorporate monomorphic sites. Assuming SNPs are pruned to one per locus, the number of monomorphic sites may be re‐scaled given its ratio to the total number of SNPs. A prior for mutation rates must then be applied as well, which may result in this same identifiability issue if it likewise exceeds one order of magnitude. For this reason, users are advised to calculate population genetic summary statistics beforehand to assess the risk of incorporating taxa that vary to such extreme degrees as to falsely signal synchrony (Figure 2), which may be exacerbated with extremely phylogenetically distant taxa. For example, if the range in ratio of monomorphic to polymorphic sites among a multi‐taxa data set greatly exceeds one order of magnitude, then extra considerations may need to be taken.

### 4.4 Informing hierarchical co‐demographic model

When conducting a multi‐taxa co‐demographic analysis using multi‐dice, the user is expected to assume a priori the composition of the demographic scenarios within the data set with respect to number of expanders and contractors, as well as accompanying prior distributions (Figure 2). Furthermore, the aSFS requires that all single‐population SFS are at the same sampling level of individuals. This can be easily accomplished with the program δaδi (Gutenkunst et al., 2009), but considering that multi‐taxa data sets usually do not consist of a uniform sampling level, an optimal sampling projection must be selected. This optimal sampling projection is typically not readily apparent as the number of SNPs varies at different projection levels, with more SNPs discarded at higher sampling projections due to missing data and decreased singleton resolution at lower sampling projections resulting in low‐frequency SNPs being assigned as monomorphic. Hence, to determine the optimal sampling projection across all taxa given this interplay between sampling of individuals and SNPs, as well as infer demographic scenarios with reasonable priors, an initial model‐based investigation can be performed for each single‐population taxon separately. While this may be performed with CL‐based methods such as δaδi (Gutenkunst et al., 2009) or fastsimcoal2 (Excoffier et al., 2013), an exploratory analysis across many independent taxa can be more efficiently conducted with an ABC approach, which allows quick inference for multiple empirical data sets against a single reference table and provides posterior distributions simultaneously with point estimates. multi‐dice coupled with an ABC framework then is well suited for efficiently performing a high throughput of such single‐population analyses to test models of demographic scenarios, explore various prior distributions and employ several data sampling levels/projections. Notably, such a preliminary analysis may also be informative for multi‐population demographic models, as well as elucidating results of synchrony from a co‐demographic analysis by identifying candidate taxa potentially involved with synchronous pulses.

## 5 CONCLUSION

The multi‐dice software package is designed for comparative population genetics and phylogeography and offers flexibility in user specification of hierarchical co‐demographic models within a command‐line interface R environment, a popular scripting language for population genetics (Paradis et al., 2017). This includes operating at different hierarchical levels (i.e., Ψ/ψ and ζ_*T*_/ζ/ζ_*s*_), applying various demographic trajectories (including co‐expansion and co‐contraction) and implementing buffering on parameter values in prior space (β), for either genomic‐scale or single‐locus sequence data. Furthermore, there are several other features not discussed here that are available in multi‐dice, such as partitioning taxa into different modelling and data specifications within a combined analysis (Prates et al., 2016). Additionally, there exist options that offer greater flexibility within the co‐demographic modelling, including incorporating two‐event/three‐epoch size change models, employing exponential rather than instantaneous growth and detecting congruence in other demographic parameters. This flexibility extends to data content and format as well, as multi‐dice also allows exploiting ancient samples, incorporating generation time heterogeneity, using polarized data (i.e., unfolded SFS), removing/adding allele frequency classes (e.g., avoiding classes more prone to error such as singletons, or including monomorphic sites and thus mutation rates and whole‐locus information), and operating simulations under fastsimcoal2's SNP model instead of its FREQ setting. Moreover, prior distributions can be highly customized, for example assigning different prior distributions between taxa within a shared pulse and those that are idiosyncratic, allocating alternative prior distributions per shared pulse and conditional buffering through a customized user‐written function that allows the β value to change depending on the prior draw rather than remain a static value across the parameter range. In consideration of this wide range of potential applications, we emphasize that as in any modelling exercise, iterative exploration is likely necessary with multi‐dice and should be embraced when it is required. We anticipate that multi‐dice will be a valuable and convenient tool for comparative population geneticists and phylogeographers.

## DATA ACCESSIBILITY

Simulation results have been deposited in Dryad (https://doi.org/10.5061/dryad.77p06). The program and user manual are available on *github*.

## AUTHOR CONTRIBUTIONS

A.T.X. performed research and software development. A.T.X. and M.J.H. designed research and wrote the manuscript.

## References

[men12686-bib-0001] Aldous, D. J. (1985). Exchangeability and related topics. Berlin, Heidelberg: Springer.

[men12686-bib-0002] Arbogast, B. S. , & Kenagy, G. J. (2001). Comparative phylogeography as an integrative approach to historical biogeography. Journal of Biogeography, 28, 819–825.

[men12686-bib-0003] Avise, J. C. (2000). Phylogeography: The history and formation of species (p. 447). Cambridge, MA: Harvard University Press.

[men12686-bib-0004] Beaumont, M. A. (2010). Approximate Bayesian computation in evolution and ecology. Annual Review of Ecology, Evolution, and Systematics, 41, 379–406.

[men12686-bib-0005] Bertorelle, G. , Benazzo, A. , & Mona, S. (2010). ABC as a flexible framework to estimate demography over space and time: Some cons, many pros. Molecular Ecology, 19, 2609–2625. 20561199 10.1111/j.1365-294X.2010.04690.x

[men12686-bib-0006] Blei, D. M. , Griffiths, T. L. , Jordan, M. I. , & Tenenbaum, J. B. (2003). Hierarchical topic models and the nested Chinese restaurant process. In: Advances in neural information processing systems (pp. 17–24). Cambridge, MA: MIT Press.

[men12686-bib-0007] Boulesteix, A.‐L. , & Strimmer, K. (2007). Partial least squares: A versatile tool for the analysis of high‐dimensional genomic data. Briefings in Bioinformatics, 8, 32–44. 16772269 10.1093/bib/bbl016

[men12686-bib-0008] Boyko, A. R. , Quignon, P. , Li, L. , Schoenebeck, J. J. , Degenhardt, J. D. , Lohmueller, K. E. , … Ostrander, E. A. (2010). A simple genetic architecture underlies morphological variation in dogs. PLoS Biology, 8, e1000451. 20711490 10.1371/journal.pbio.1000451 PMC2919785

[men12686-bib-0009] Burbrink, F. T. , Chan, Y. L. , Myers, E. A. , Ruane, S. , Smith, B. T. , & Hickerson, M. J. (2016). Asynchronous demographic responses to Pleistocene climate change in Eastern Nearctic vertebrates. Ecology Letters, 19, 1457–1467. 27781365 10.1111/ele.12695

[men12686-bib-0010] Bustamante, C. D. , Wakeley, J. , Sawyer, S. , & Hartl, D. L. (2001). Directional selection and the site‐frequency spectrum. Genetics, 159, 1779–1788. 11779814 10.1093/genetics/159.4.1779 PMC1461920

[men12686-bib-0011] Carnaval, A. C. , Hickerson, M. J. , Haddad, C. F. B. , Rodrigues, M. T. , & Moritz, C. (2009). Stability predicts genetic diversity in the Brazilian Atlantic Forest hotspot. Science, 323, 785–789. 19197066 10.1126/science.1166955

[men12686-bib-0012] Carstens, B. C. , Gruenstaeudl, M. , & Reid, N. M. (2016). Community trees: Identifying codiversification in the Páramo dipteran community. Evolution, 70, 1080–1093. 27061575 10.1111/evo.12916

[men12686-bib-0013] Chan, Y. L. , Schanzenbach, D. , & Hickerson, M. J. (2014). Detecting concerted demographic response across community assemblages using hierarchical approximate Bayesian computation. Molecular Biology and Evolution, 31, 2501–2515. 24925925 10.1093/molbev/msu187 PMC4137712

[men12686-bib-0014] Congdon, P. (2001). Bayesian statistical modelling. Chichester, West Sussex: John Wiley & Sons.

[men12686-bib-0015] Csilléry, K. , François, O. , & Blum, M. G. B. (2012). abc: An R package for approximate Bayesian computation (ABC). Methods in Ecology and Evolution, 3, 475–479. 10.1016/j.tree.2010.04.001 20488578

[men12686-bib-0016] Díaz‐Uriarte, R. , & Alvarez de Andrés, S. (2006). Gene selection and classification of microarray data using random forest. BMC Bioinformatics, 7, 3. 16398926 10.1186/1471-2105-7-3 PMC1363357

[men12686-bib-0017] Ewing, G. , Hermisson, J. (2010) MSMS: A coalescent simulation program including recombination, demographic structure and selection at a single locus. Bioinformatics (Oxford, England), 26, 2064–5. 10.1093/bioinformatics/btq322 PMC2916717 20591904

[men12686-bib-0018] Excoffier, L. , Dupanloup, I. , Huerta‐Sánchez, E. , Sousa, V. C. , & Foll, M. (2013). Robust demographic inference from genomic and SNP data. PLoS Genetics, 9, e1003905. 24204310 10.1371/journal.pgen.1003905 PMC3812088

[men12686-bib-0019] Fouquet, A. , Noonan, B. P. , Rodrigues, M. T. , Pech, N. , Gilles, A. , & Gemmell, N. J. (2012). Multiple quaternary refugia in the eastern guiana shield revealed by comparative phylogeography of 12 frog species. Systematic Biology, 61, 461–489. 22223446 10.1093/sysbio/syr130

[men12686-bib-0020] Frantz, L. A. F. , Schraiber, J. G. , Madsen, O. , Megens, H.‐J. , Cagan, A. , Bosse, M. , … Groenen, M. A. M. (2015). Evidence of long‐term gene flow and selection during domestication from analyses of Eurasian wild and domestic pig genomes. Nature Genetics, 47, 1141–1148. 26323058 10.1038/ng.3394

[men12686-bib-0021] Frichot, E. , Mathieu, F. , Trouillon, T. , Bouchard, G. , & François, O. (2014). Fast and efficient estimation of individual ancestry coefficients. Genetics, 196, 973–83. 24496008 10.1534/genetics.113.160572 PMC3982712

[men12686-bib-0022] Gelman, A. , Carlin, J. B. , Stern, H. S. , & Rubin, D. B. (2003). Bayesian Data Analysis. Boca Raton, FL: Chapman & Hall/CRC.

[men12686-bib-0023] Gignoux, C. R. , Henn, B. M. , & Mountain, J. L. (2011). Rapid, global demographic expansions after the origins of agriculture. Proceedings of the National Academy of Sciences of the United States of America, 108, 6044–6049. 21444824 10.1073/pnas.0914274108 PMC3076817

[men12686-bib-0024] Gutenkunst, R. N. , Hernandez, R. D. , Williamson, S. H. , & Bustamante, C. D. (2009). Inferring the joint demographic history of multiple populations from multidimensional SNP frequency data. PLoS Genetics, 5, e1000695. 19851460 10.1371/journal.pgen.1000695 PMC2760211

[men12686-bib-0025] He, D. , Chen, Y. , Liu, C. , Tao, J. , Ding, C. , & Chen, Y. (2016). Comparative phylogeography and evolutionary history of schizothoracine fishes in the Changtang Plateau and their implications for the lake level and Pleistocene climate fluctuations. Ecology and Evolution, 6, 656–674. 26865956 10.1002/ece3.1890 PMC4739559

[men12686-bib-0026] Hewitt, G. M. (1996). Some genetic consequences of ice ages, and their role in divergence and speciation. Biological Journal of the Linnean Society, 58, 247–276.

[men12686-bib-0027] Hewitt, G. (2000). The genetic legacy of the quaternary ice ages. Nature, 405, 907–913. 10879524 10.1038/35016000

[men12686-bib-0028] Hickerson, M. J. , Carstens, B. C. , Cavender‐Bares, J. , Crandall, K. A. , Graham, C. H. , Johnson, J. B. , … Yoder, A. D. (2010). Phylogeography's past, present, and future: 10 years after Avise, 2000. Molecular Phylogenetics and Evolution, 54, 291–301. 19755165 10.1016/j.ympev.2009.09.016

[men12686-bib-0029] Hickerson, M. J. , Dolman, G. , & Moritz, C. (2006). Comparative phylogeographic summary statistics for testing simultaneous vicariance. Molecular Ecology, 15, 209–23. 16367841 10.1111/j.1365-294X.2005.02718.x

[men12686-bib-0030] Hickerson, M. J. , Stahl, E. , & Takebayashi, N. (2007). msBayes: Pipeline for testing comparative phylogeographic histories using hierarchical approximate Bayesian computation. BMC Bioinformatics, 8, 268. 17655753 10.1186/1471-2105-8-268 PMC1949838

[men12686-bib-0031] Hickerson, M. J. , Stone, G. N. , Lohse, K. , Demos, T. C. , Xie, X. , Landerer, C. , & Takebayashi, N. (2014). Recommendations for using msBayes to incorporate uncertainty in selecting an ABC model prior: A response to Oaks et al. Evolution, 68, 284–294. 24102483 10.1111/evo.12241

[men12686-bib-0032] Hohenlohe, P. A. , Bassham, S. , Etter, P. D. , Stiffler, N. , Johnson, E. A. , & Cresko, W. A. (2010). Population genomics of parallel adaptation in threespine stickleback using sequenced RAD tags. PLoS Genetics, 6, e1000862. 20195501 10.1371/journal.pgen.1000862 PMC2829049

[men12686-bib-0033] Huang, W. , Takebayashi, N. , Qi, Y. , & Hickerson, M. J. (2011). MTML‐msBayes: Approximate Bayesian comparative phylogeographic inference from multiple taxa and multiple loci with rate heterogeneity. BMC Bioinformatics, 12, 1. 21199577 10.1186/1471-2105-12-1 PMC3031198

[men12686-bib-0034] Kamm, J. A. , Terhorst, J. , & Song, Y. S. (2017). Efficient computation of the joint sample frequency spectra for multiple populations. Journal of Computational and Graphical Statistics, 26, 182–194. 28239248 10.1080/10618600.2016.1159212 PMC5319604

[men12686-bib-0035] Kautt, A. F. , Machado‐Schiaffino, G. , & Meyer, A. (2016). Multispecies outcomes of sympatric speciation after admixture with the source population in two radiations of Nicaraguan Crater Lake Cichlids. PLoS Genetics, 12, e1006157. 27362536 10.1371/journal.pgen.1006157 PMC4928843

[men12686-bib-0036] Keinan, A. , & Clark, A. G. (2012). Recent explosive human population growth has resulted in an excess of rare genetic variants. Science, 336, 740–3. 22582263 10.1126/science.1217283 PMC3586590

[men12686-bib-0037] Kern, A. D. , & Schrider, D. R. (2016). Discoal: Flexible coalescent simulations with selection. Bioinformatics, 32, 3839–3841. 27559153 10.1093/bioinformatics/btw556 PMC5167068

[men12686-bib-0038] Lemaire, L. , Jay, F. , Lee, I.‐H. , Csilléry, K. , & Blum, M. G. B. (2016). Goodness‐of‐fit statistics for approximate Bayesian computation. arXiv, eprint arXiv: 1601.04096.

[men12686-bib-0039] Liaw, A. , & Wiener, M. (2002). Classification and regression by randomForest. R News, 2, 18–22.

[men12686-bib-0040] Liu, D. C. , & Nocedal, J. (1989). On the limited memory BFGS method for large scale optimization. Mathematical Programming, 45, 503–528.

[men12686-bib-0041] Lopes, J. S. , & Beaumont, M. A. (2010). ABC: A useful Bayesian tool for the analysis of population data. Infection, Genetics and Evolution, 10, 825–832. 10.1016/j.meegid.2009.10.010 19879976

[men12686-bib-0042] Lukic, S. , & Hey, J. (2012). Demographic inference using spectral methods on SNP data, with an analysis of the human out‐of‐Africa expansion. Genetics, 192, 619–639. 22865734 10.1534/genetics.112.141846 PMC3454885

[men12686-bib-0043] Luo, D. , Yue, J. P. , Sun, W. G. , Xu, B. , Li, Z. M. , Comes, H. P. , & Sun, H. (2015). Evolutionary history of the subnival flora of the Himalaya‐Hengduan Mountains: First insights from comparative phylogeography of four perennial herbs. Journal of Biogeography, 43, 31–43.

[men12686-bib-0044] Massatti, R. , & Knowles, L. L. (2016). Contrasting support for alternative models of genomic variation based on microhabitat preference: Species‐specific effects of climate change in alpine sedges. Molecular Ecology, 25, 3974–3986. 27317885 10.1111/mec.13735

[men12686-bib-0045] Mazet, O. , Rodríguez, W. , Grusea, S. , Boitard, S. , & Chikhi, L. (2016). On the importance of being structured: Instantaneous coalescence rates and human evolution‐lessons for ancestral population size inference? Heredity, 116, 362–371. 26647653 10.1038/hdy.2015.104 PMC4806692

[men12686-bib-0046] Mevik, B.‐H. , & Wehrens, R. (2007). The pls package: Principal component and partial least squares regression in R. Journal of Statistical Software, 18, 1–24.

[men12686-bib-0047] Nadachowska‐Brzyska, K. , Li, C. , Smeds, L. , Zhang, G. , & Ellegren, H. (2015). Temporal dynamics of avian populations during pleistocene revealed by whole‐genome sequences. Current Biology, 25, 1375–1380. 25891404 10.1016/j.cub.2015.03.047 PMC4446789

[men12686-bib-0048] Oaks, J. R. (2014). An improved approximate‐Bayesian model‐choice method for estimating shared evolutionary history. BMC Evolutionary Biology, 14, 150. 24992937 10.1186/1471-2148-14-150 PMC4227068

[men12686-bib-0049] Ornelas, J. F. , Sosa, V. , Soltis, D. E. , Daza, J. M. , González, C. , Soltis, P. S. , … Ruiz‐Sanchez, E. (2013). Comparative phylogeographic analyses illustrate the complex evolutionary history of threatened cloud forests of Northern Mesoamerica. PLoS ONE, 8, e56283. 23409165 10.1371/journal.pone.0056283 PMC3567015

[men12686-bib-0050] Papadopoulou, A. , & Knowles, L. L. (2015). Species‐specific responses to island connectivity cycles: Refined models for testing phylogeographic concordance across a Mediterranean Pleistocene aggregate island complex. Molecular Ecology, 24, 4252–4268. 26154606 10.1111/mec.13305

[men12686-bib-0051] Papadopoulou, A. , & Knowles, L. L. (2016). Toward a paradigm shift in comparative phylogeography driven by trait‐based hypotheses. Proceedings of the National Academy of Sciences of the United States of America, 113, 8018–24. 27432974 10.1073/pnas.1601069113 PMC4961141

[men12686-bib-0052] Paradis, E. , Gosselin, T. , Grünwald, N. J. , Jombart, T. , Manel, S. , & Lapp, H. (2017). Towards an integrated ecosystem of R packages for the analysis of population genetic data. Molecular Ecology Resources, 17, 1–4. 27860406 10.1111/1755-0998.12636

[men12686-bib-0053] Patterson, N. , Price, A. L. , & Reich, D. (2006). Population structure and eigenanalysis. PLoS Genetics, 2, e190. 17194218 10.1371/journal.pgen.0020190 PMC1713260

[men12686-bib-0054] Poh, Y.‐P. , Domingues, V. S. , Hoekstra, H. E. , & Jensen, J. D. (2014). On the prospect of identifying adaptive loci in recently bottlenecked populations. PLoS ONE, 9, e110579. 25383711 10.1371/journal.pone.0110579 PMC4226487

[men12686-bib-0055] Prates, I. , Xue, A. T. , Brown, J. L. , Alvarado‐Serrano, D. F. , Rodrigues, M. T. , Hickerson, M. J. , & Carnaval, A. C. (2016). Inferring responses to climate dynamics from historical demography in neotropical forest lizards. Proceedings of the National Academy of Sciences, 113, 7978–7985. 10.1073/pnas.1601063113 PMC4961184 27432951

[men12686-bib-0056] Pudlo, P. , Marin, J.‐M. , Estoup, A. , Cornuet, J.‐M. , Gauthier, M. , & Robert, C. P. (2016). Reliable ABC model choice via random forests. Bioinformatics, 32, 859–866. 26589278 10.1093/bioinformatics/btv684

[men12686-bib-0057] Qian, S. S. , Donnelly, M. , Schmelling, D. C. , Messner, M. , Linden, K. G. , & Cotton, C. (2004). Ultraviolet light inactivation of protozoa in drinking water: A Bayesian meta‐analysis. Water Research, 38, 317–26. 14675643 10.1016/j.watres.2003.10.007

[men12686-bib-0058] Qu, Y. , Song, G. , Gao, B. , Quan, Q. , Ericson, P. G. P. , & Lei, F. (2015). The influence of geological events on the endemism of East Asian birds studied through comparative phylogeography. Journal of Biogeography, 42, 179–192.

[men12686-bib-0059] Rannala, B. (2015). The art and science of species delimitation. Current Zoology, 61, 846–853.

[men12686-bib-0060] Robinson, J. D. , Coffman, A. J. , Hickerson, M. J. , & Gutenkunst, R. N. (2014). Sampling strategies for frequency spectrum‐based population genomic inference. BMC Evolutionary Biology, 14, 254. 25471595 10.1186/s12862-014-0254-4 PMC4269862

[men12686-bib-0061] Rougemont, Q. , Gagnaire, P.‐A. , Perrier, C. , Genthon, C. , Besnard, A.‐L. , Launey, S. , & Evanno, G. (2017). Inferring the demographic history underlying parallel genomic divergence among pairs of parasitic and non‐parasitic lamprey ecotypes. Molecular Ecology, 26, 142–162. 27105132 10.1111/mec.13664

[men12686-bib-0062] Rougeux, C. , Bernatchez, L. , & Gagnaire, P.‐A. (2016). Modeling the multiple facets of speciation‐with‐gene‐flow towards improving divergence history inference of a recent fish adaptive radiation. bioRxiv, 68932.

[men12686-bib-0063] Sawyer, S. A. , & Hartl, D. L. (1992). Population genetics of polymorphism and divergence. Genetics, 132, 1161–1176. 1459433 10.1093/genetics/132.4.1161 PMC1205236

[men12686-bib-0064] Smith, B. T. , McCormack, J. E. , Cuervo, A. M. , Hickerson, M. J. , Aleixo, A. , Cadena, C. D. , … Brumfield, R. T. (2014). The drivers of tropical speciation. Nature, 515, 406–409. 25209666 10.1038/nature13687

[men12686-bib-0065] Stone, G. N. , Lohse, K. , Nicholls, J. A. , Fuentes‐Utrilla, P. , Sinclair, F. , Schönrogge, K. , … Hickerson, M. J. (2012). Reconstructing community assembly in time and space reveals enemy escape in a Western Palearctic insect community. Current Biology, 22, 532–7. 22405865 10.1016/j.cub.2012.01.059

[men12686-bib-0066] Strobl, C. , Boulesteix, A.‐L. , Zeileis, A. , & Hothorn, T. (2007). Bias in random forest variable importance measures: Illustrations, sources and a solution. BMC Bioinformatics, 8, 1. 17254353 10.1186/1471-2105-8-25 PMC1796903

[men12686-bib-0067] Svetnik, V. , Liaw, A. , Tong, C. , Culberson, J. C. , Sheridan, R. P. , & Feuston, B. P. (2003). Random forest: A classification and regression tool for compound classification and QSAR modeling. Journal of Chemical Information and Computer Sciences, 43, 1947–1958. 14632445 10.1021/ci034160g

[men12686-bib-0068] Taberlet, P. , Fumagalli, L. , Wust‐Saucy, A.‐G. , & Cosson, J.‐F. (1998). Comparative phylogeography and postglacial colonization routes in Europe. Molecular Ecology, 7, 453–464. 9628000 10.1046/j.1365-294x.1998.00289.x

[men12686-bib-0069] Wakeley, J. , & Hey, J. (1997). Estimating ancestral population parameters. Genetics, 145, 847–855. 9055093 10.1093/genetics/145.3.847 PMC1207868

[men12686-bib-0070] Wegmann, D. , Leuenberger, C. , & Excoffier, L. (2009). Efficient approximate Bayesian computation coupled with Markov chain Monte Carlo without likelihood. Genetics, 182, 1207–1218. 19506307 10.1534/genetics.109.102509 PMC2728860

[men12686-bib-0071] Wood, D. A. , Vandergast, A. G. , Barr, K. R. , Inman, R. D. , Esque, T. C. , Nussear, K. E. , & Fisher, R. N. (2013). Comparative phylogeography reveals deep lineages and regional evolutionary hotspots in the Mojave and Sonoran deserts. Diversity and Distributions, 19, 722–737.

[men12686-bib-0072] Xue, A. T. , & Hickerson, M. J. (2015). The aggregate site frequency spectrum for comparative population genomic inference. Molecular Ecology, 24, 6223–6240. 26769405 10.1111/mec.13447 PMC4717917

