# Supplementary material for: multi‐dice: r package for comparative population genomic inference under hierarchical co‐demographic models of independent single‐population size changes
Source: Mol Ecol Resour. 2017 May 30;17(6):e212–24. doi: 10.1111/1755-0998.12686 (PMC5724483; doi:10.1111/1755-0998.12686)
Supplement: Supplementary file 1 [file MEN-17-e212-s001.docx]

***Multi-DICE*: R package for comparative population genomic inference under hierarchical co-demographic models of independent single-population size changes**

Alexander T. Xue^1^ (ATX) and Michael J. Hickerson^1,2^ (MJH)

^1^ Department of Biology: Subprogram in Ecology, Evolutionary Biology, and Behavior, City College and Graduate Center of City University of New York, 160 Convent Avenue, Marshak Science Building, Room 526, New York, NY 10031

^2^ Division of Invertebrate Zoology, American Museum of Natural History, Central Park West at 79^th^ Street, New York, NY 10024

*Testing Inferential Frameworks Extended Methods*

To generate PODs and reference tables, we employed the hyperprior ζ*_s_* ~ *U*{2, (10–2*(Ψ–1))}/10, such that each synchronous pulse had at least two taxa. Single-population SFS were produced from 25,000 independent genealogies and following the prior distributions τ ~ *U*{5,000, 200,000} (in units of number of generations), ε_τ_*_s_* (ε for taxa within synchronous pulses) ~ ln *U*(0.01, 0.10) for co-expanders, ε_τ_*_s_* ~ 1/ln *U*(0.01, 0.10) for co-contractors, ε_τ_*_i_* (ε for idiosyncratic taxa) ~ ln *U*(0.01, 1.00) for co-expanders, ε_τ_*_i_* ~ 1/ln *U*(0.01, 1.00) for co-contractors, and *N* ~ *U*{50,000, 250,000}. Importantly, in the special case of ψ = 0, since here Ψ = 10 and all other values of ψ result in Ψ < 10, which results in 0 acting as a poor numeric representation of this scenario for estimation purposes, all instances of ψ = 0 were converted to Ψ = 10.

To achieve hCL inference, a custom Python protocol was employed that calls from *dadi* to calculate the expected SFS, deploys the multinomially distributed CL equation applied in *fastsimcoal2*, and operates the BFGS optimization algorithm as done in *dadi*. Importantly, instead of obtaining the expected SFS through stochastic coalescent simulations from *fastsimcoal2*, we selected *dadi* to derive a deterministic expected SFS from its Wright-Fischer-based diffusion approximation algorithm and accordingly used the BFGS algorithm. The latter should not meaningfully deviate from the former for single-population SFS, yet its deterministic nature may be more appropriate for exploring the increased complexity in parameter and thus likelihood space associated with our hierarchical co-demographic model. To accommodate parameter hierarchy, each discrete value within the ψ hyperprior was treated as a separate model and the vectors ζ and τ*_s_* were subsequently optimized given initially-drawn and fixed nuisance taxon-specific demographic parameter values (*i.e.* τ*_i_*, *N*, and ε across all taxa). There were 200 optimization replicates per ψ model, for a total of 1200 replicates per POD inference, and ψ estimation was based on the replicate with the highest likelihood among the total set. We selected 200 replicates per ψ value to balance between sufficiently exploring the hierarchical levels of likelihood hyperspace and practical considerations of computational expense. To clarify, each replicate had an *a priori* assignment of taxon-specific parameter values that was independent from all other replicates and remained constant throughout optimization.

There existed a swapping tradeoff among τ*_s_* and τ*_i_* values as the values in the vector ζ*_s_* changed throughout optimization. In other words, as optimization progressed, the assignment of τ*_s_* and τ*_i_* values relative to the initially-drawn and fixed *N* and ε values changed correspondingly. For example, if a particular replicate given *n* = 10 and ψ = 2 changes during an optimization step from ζ_1_ = 0.6 and ζ_2_ = 0.4 to ζ_1_ = 0.5 and ζ_2_ = 0.4, then the first six sets of fixed *N* and ε values that were previously coupled with τ*_s_*_,1_ and the next four sets of fixed *N* and ε values that were previously coupled with τ*_s_*_,2_ would be re-shuffled such that instead, to calculate the expected aSFS and associated likelihood, the first five sets of fixed *N* and ε values are coupled with τ*_s_*_,1_, the next four sets of fixed *N* and ε values are coupled with τ*_s_*_,2_, and the last tenth set of fixed *N* and ε values are coupled with the fixed τ*_i_* value when σ = 1. Importantly in this example, the sixth set of *N* and ε values changed from being coupled with τ*_s_*_,1_ to τ*_s_*_,2_, and the tenth set of *N* and ε values changed from being coupled with τ*_s_*_,2_ to the τ*_i_* value initially drawn to be assigned to the tenth set of *N* and ε values. Notably, the initially-drawn and fixed vector of {τ_(2*ψ)+1_, …, τ_10_} continuously has values displaced throughout optimization, the number of which is dependent on *S* and σ. To avoid a similar routine for ε, the broader distribution of ε ~ log *U*(0.01, 1.00) was employed for initial parameter draws, regardless of pulse membership. All other distributions for initial parameter draws and parameter search ranges were the same as the priors outlined above.

Borrowing from the CL procedure implemented in *fastsimcoal2*, optimization was performed on the difference between the expected and observed likelihood, with the rule that the expected could not exceed the observed. For each replicate, there were 40 iterative cycles of BFGS optimization, which is the standard amount for the analogous optimization method within *fastsimcoal2* and exceeds the threshold for BFGS convergence, which is the number of free parameters squared, at most ψ values (3 parameters for ζ and 3 parameters for τ*_s_* when ψ = 3 🡺 6^2^ = 36 cycles).


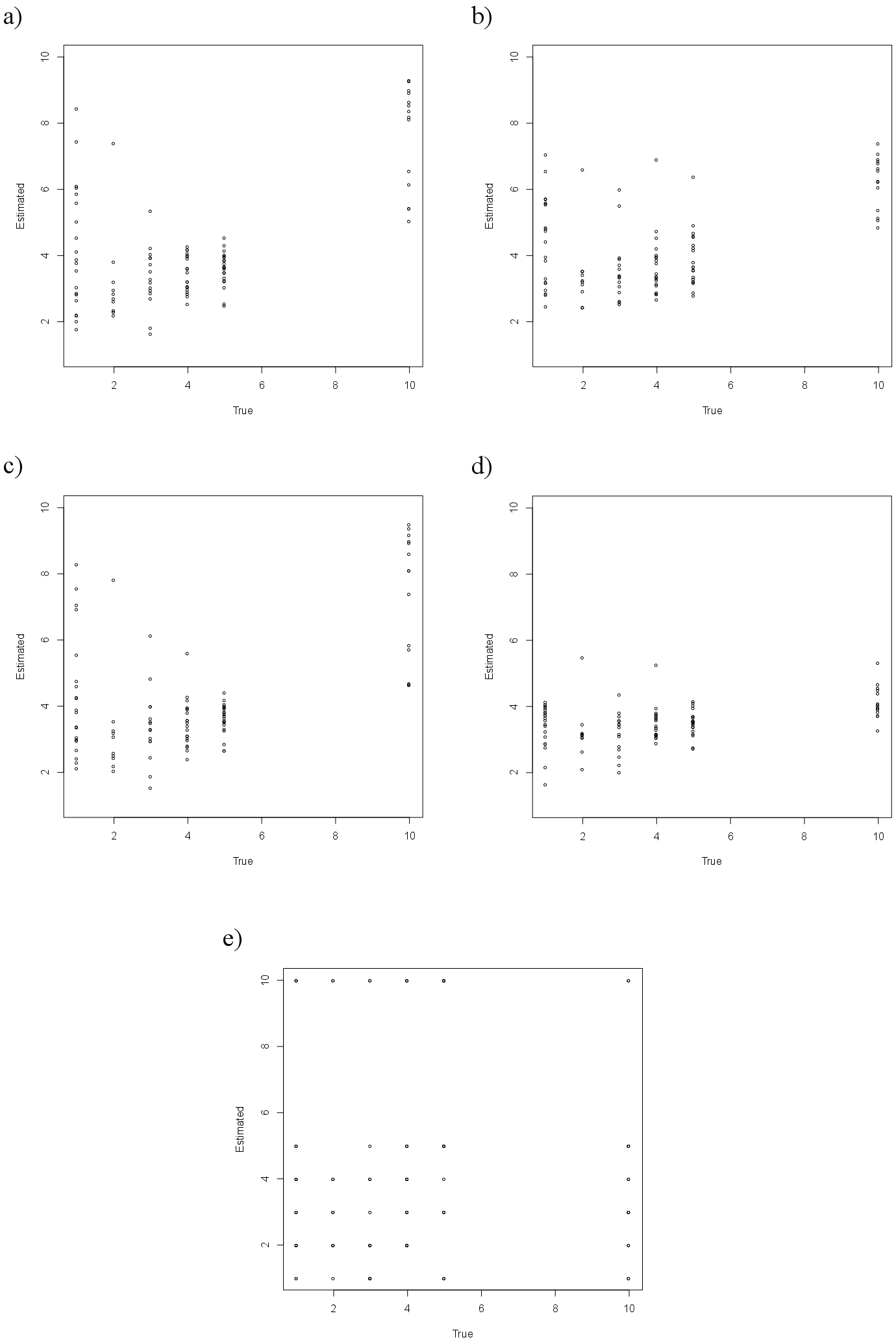


**Figure S1. Cross-validation plots for testing inferential frameworks under co-expansion model.** True Ψ values on the x-axis and estimated Ψ values on the y-axis across 100 total PODs. These plots correspond to values reported in Table 4. a) hRF; b) hRF with PLS; c) hABC; d) hABC with PLS; e) hCL. For c) and d), mean estimates given a tolerance of 0.0005 are displayed; plots of median and mode estimates are difficult to interpret due to overlapping of points resulting from discrete value estimates, and plots across tolerance levels are qualitatively similar, with 0.0005 being the most accurate. Importantly, e) demonstrates lacking signal to estimate Ψ. Furthermore, coupling with PLS in b) and d) does not appear to offer any improvement in estimation compared to a) and c), respectively. Additionally, for a) – d), PODs simulated under a true Ψ = 1 value particularly suffered from identifiability issues, such that estimates are not well correlated since these range across most possible Ψ values. This can be largely attributed to the wide flexibility in the corresponding ζ*_s_* hyperprior, which allowed a high degree of idiosyncrasy.


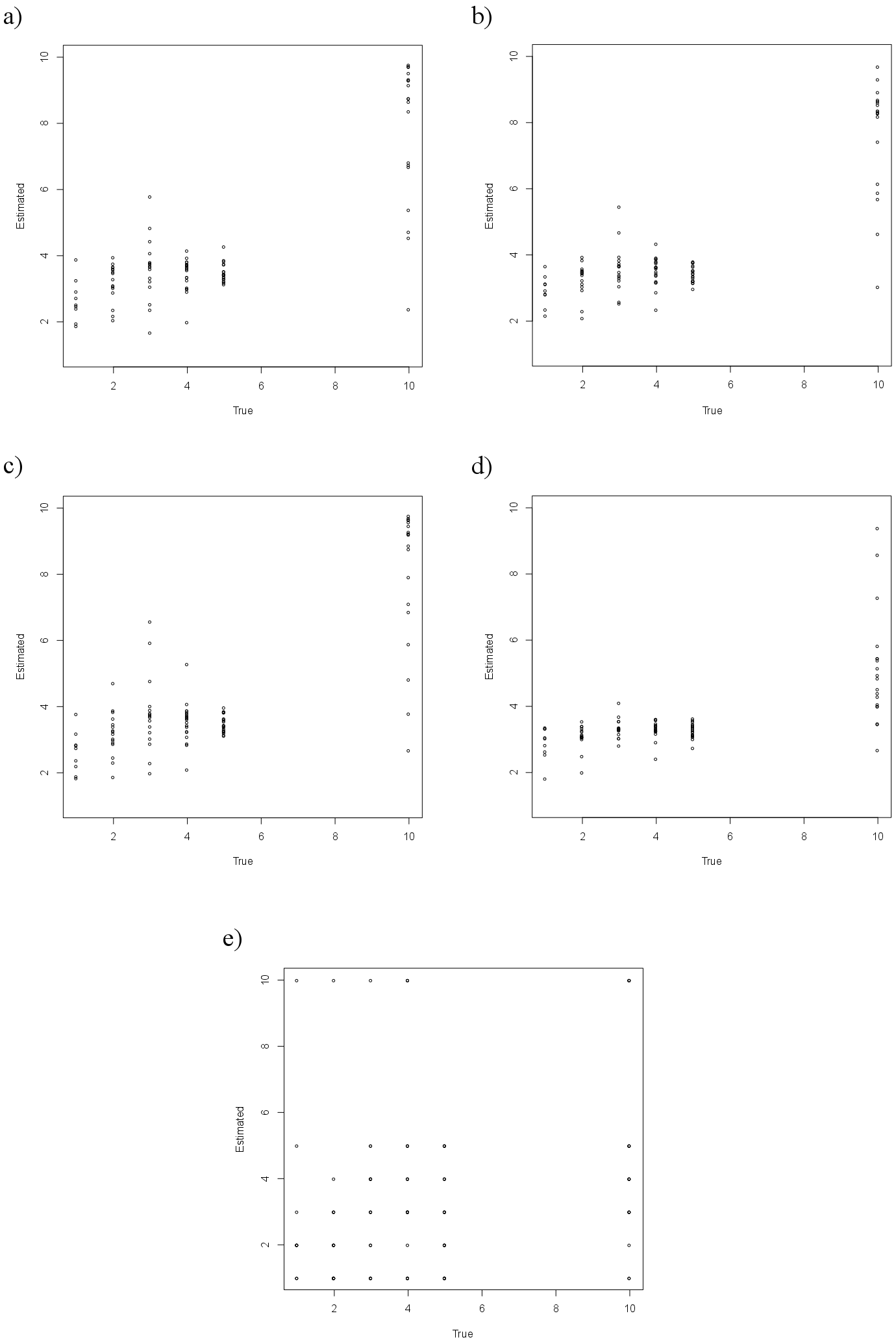


**Figure S2. Cross-validation plots for testing inferential frameworks under co-contraction model.** True Ψ values on the x-axis and estimated Ψ values on the y-axis across 100 total PODs. These plots correspond to values reported in Table 4. a) hRF; b) hRF with PLS; c) hABC; d) hABC with PLS; e) hCL. For c) and d), mean estimates given a tolerance of 0.0005 are displayed; plots of median and mode estimates are difficult to interpret due to overlapping of points resulting from discrete value estimates, and plots across tolerance levels are qualitatively similar, with 0.0005 being the most accurate. The patterns illustrated here are largely consistent with Figure S1.


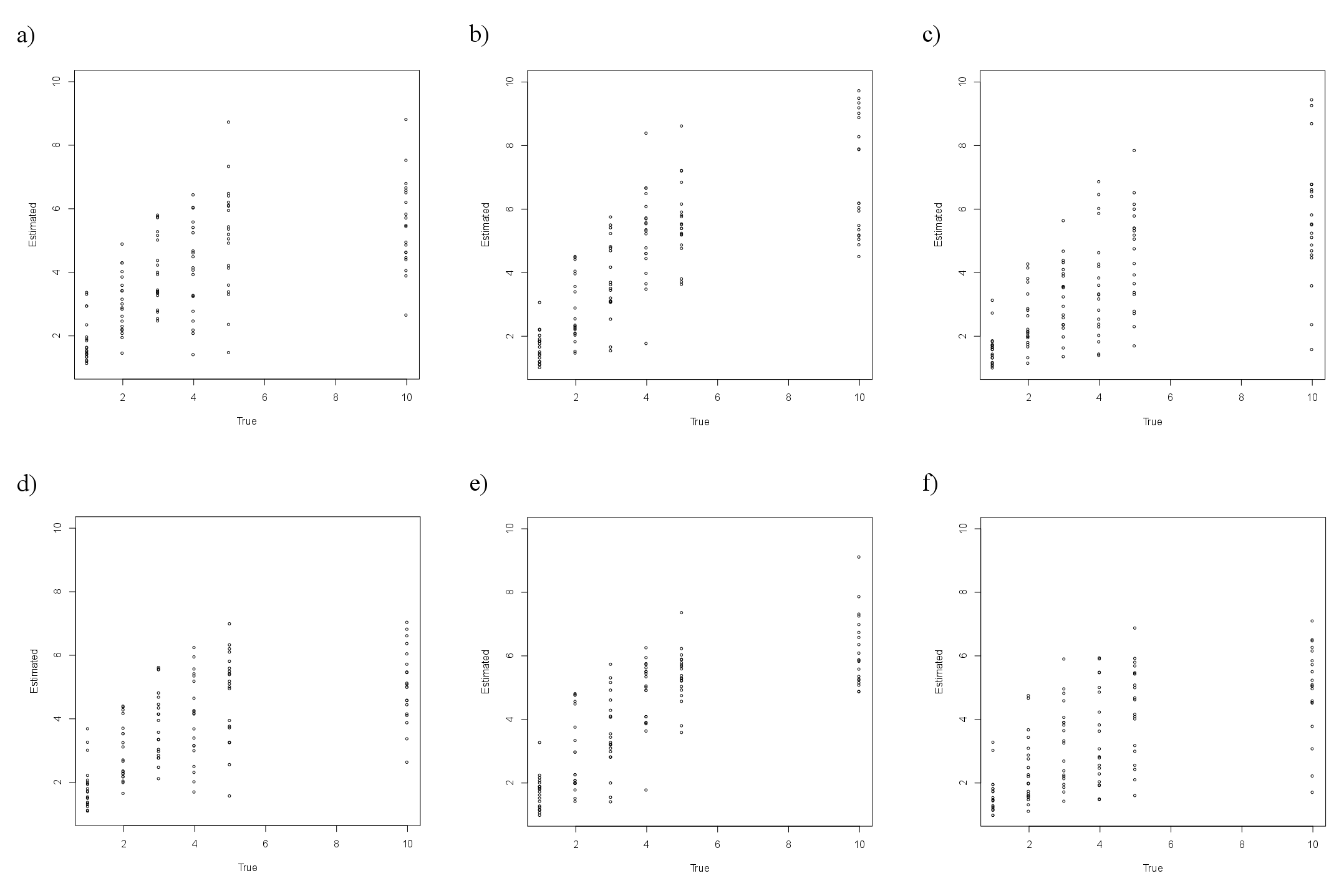


**Figure S3. Cross-validation plots for pulse buffer experiment estimating Ψ.** True Ψ values on the x-axis and estimated Ψ values on the y-axis across 20 PODs per true Ψ value for 120 total PODs. These plots correspond to values reported in Table 5. Column 1, or a) & d): β = 0; Column 2, or b) & e): β = 30,000; Column 3, or c) & f): β = 0 for PODs and β = 30,000 for reference table; Row 1, or a) – c): hRF; Row 2, or d) – f): hABC. For hABC, mean estimates are displayed, since plots of median and mode estimates are difficult to interpret due to overlapping of points resulting from discrete value estimates. Expectedly, buffer implementation seemingly had greater effect at higher Ψ values.


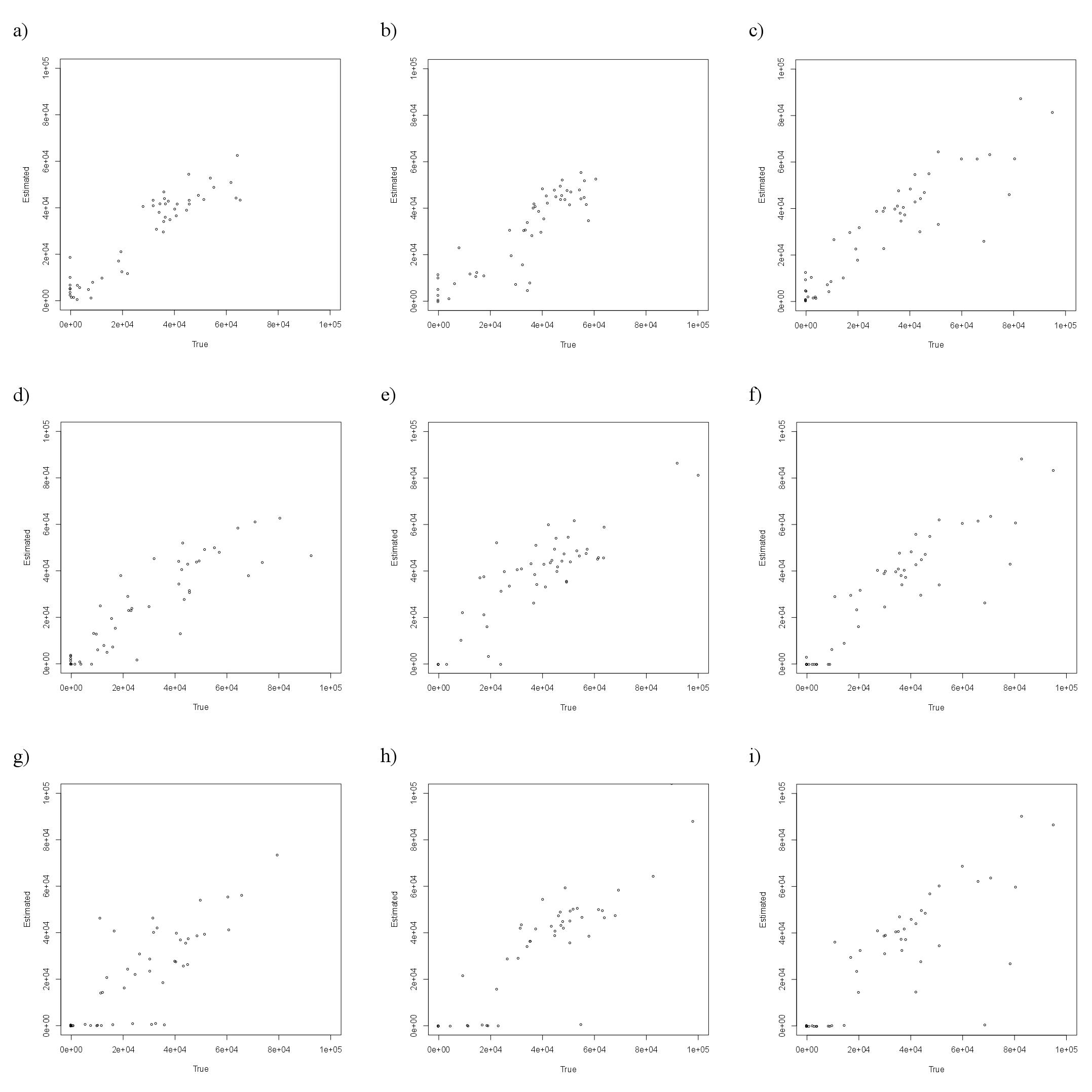


**Figure S4. Cross-validation plots for pulse buffer experiment estimating Ω.** True Ω values on the x-axis and estimated Ω values on the y-axis across 50 total PODs. These plots correspond to values reported in Table 5. Column 1, or a), d), & g): β = 0; Column 2, or b), e), & h): β = 30,000; Column 3, or c), f), & i): β = 0 for PODs and β = 30,000 for reference table; Row 1, or a) – c): hABC mean estimates; Row 2, or d) – f): hABC median estimates; Row 3, or g) – i): hABC mode estimates. Notably, buffer implementation had minimal effect on Ω estimation.


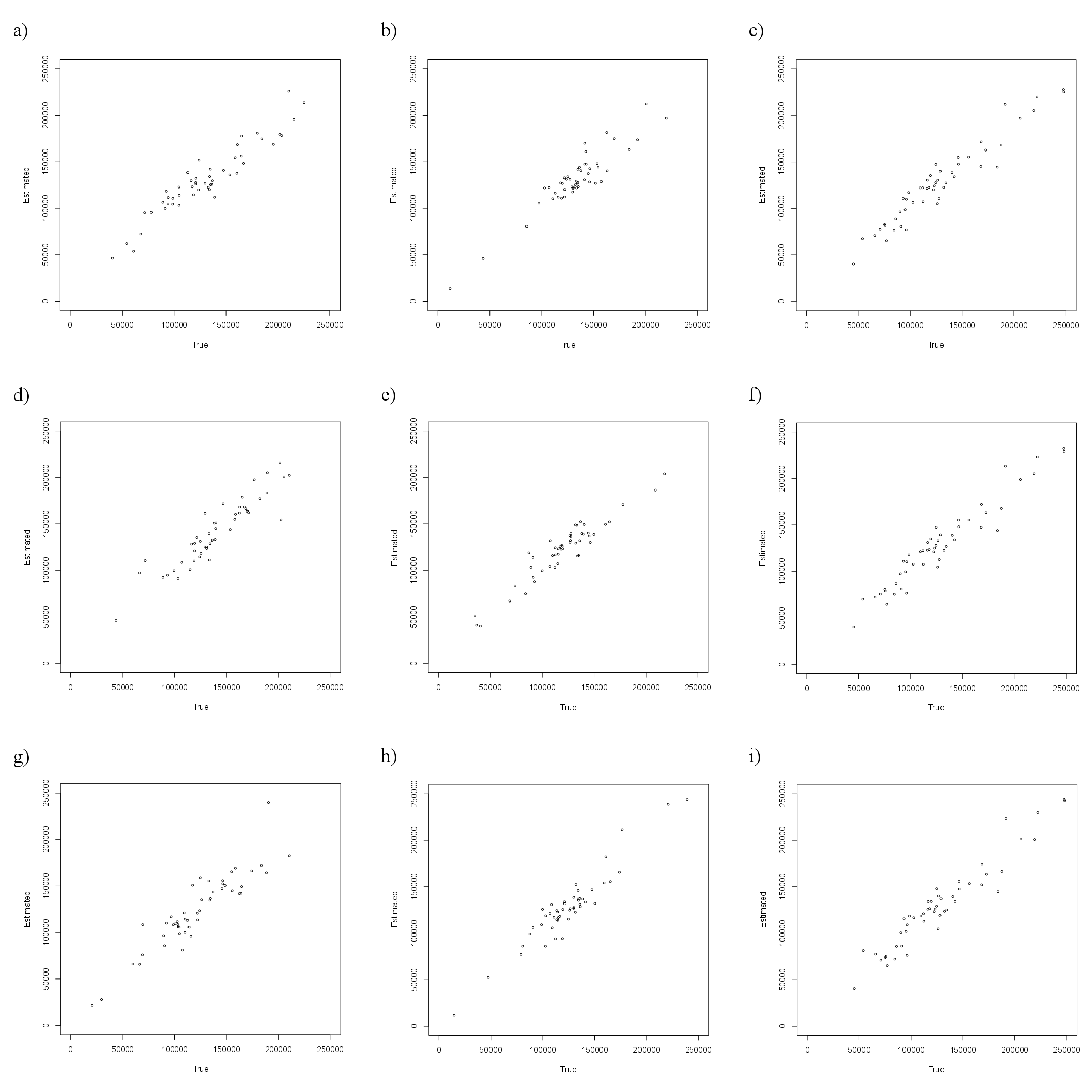


**Figure S5. Cross-validation plots for pulse buffer experiment estimating E(τ).** True E(τ) values on the x-axis and estimated E(τ) values on the y-axis across 50 total PODs. These plots correspond to values reported in Table 5. Column 1, or a), d), & g): β = 0; Column 2, or b), e), & h): β = 30,000; Column 3, or c), f), & i): β = 0 for PODs and β = 30,000 for reference table; Row 1, or a) – c): hABC mean estimates; Row 2, or d) – f): hABC median estimates; Row 3, or g) – i): hABC mode estimates. Notably, buffer implementation had minimal effect on E(τ) estimation.


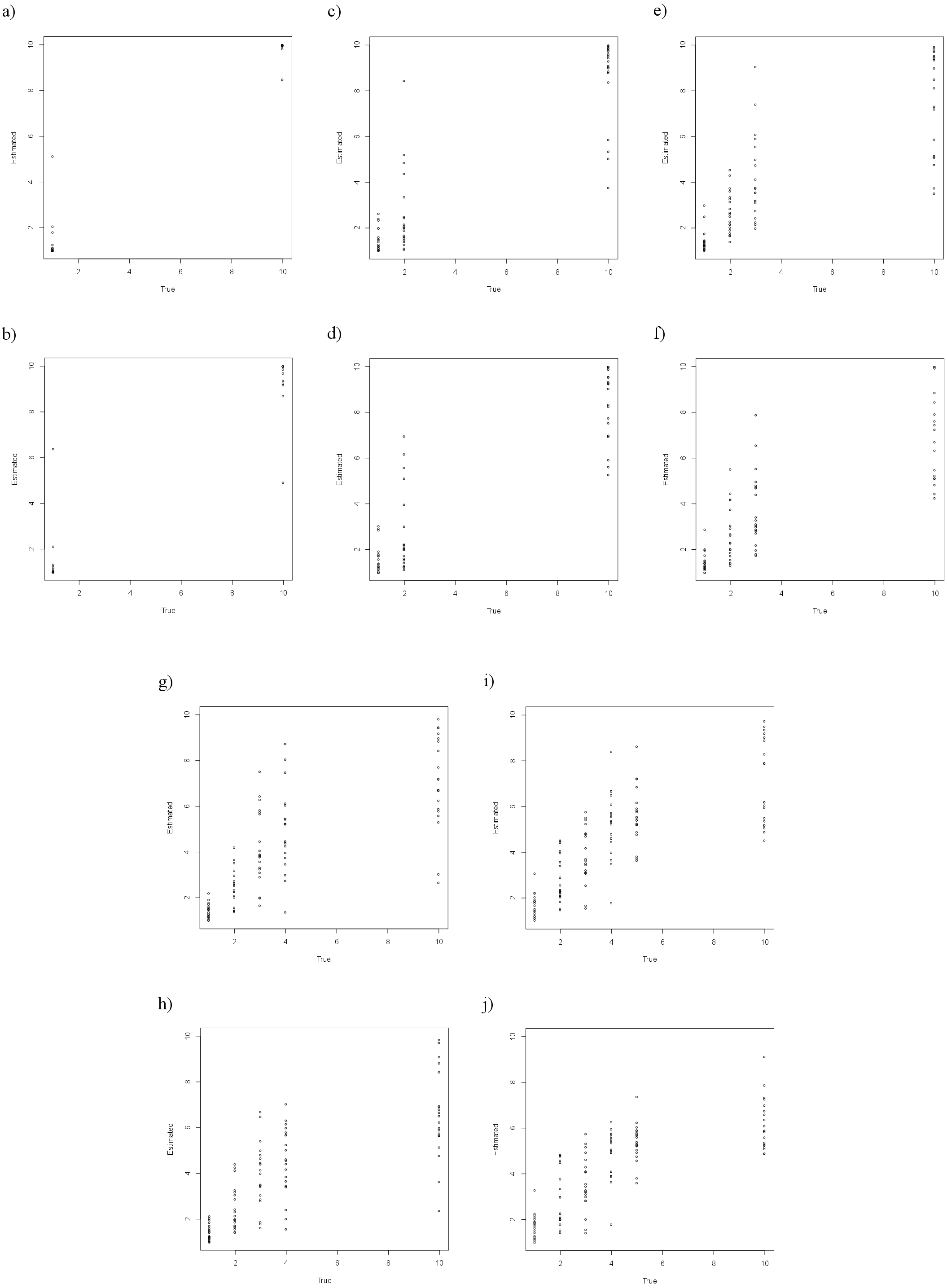


**Figure S6. Cross-validation plots for truncating Ψ hyperprior experiment.** True Ψ values on the x-axis and estimated Ψ values on the y-axis across 20 PODs per true Ψ value. These plots correspond to the values reported in Table 6. a) & b): ѱ ~ *U*{0, 1}; c) & d): ѱ ~ *U*{0, 2}; e) & f): ѱ ~ *U*{0, 3}; g) & h): ѱ ~ *U*{0, 4}; i) & j): ѱ ~ *U*{0, 5}; a), c), e), g), & i): hRF; b), d), f), h), & j): hABC. For hABC, mean estimates are displayed, since plots of median and mode estimates are difficult to interpret due to overlapping of points resulting from discrete value estimates.
